# Exploring the timeline and network interplay of immune mediators in COVID-19 patients according to disease outcome

**DOI:** 10.3389/fimmu.2026.1765997

**Published:** 2026-03-16

**Authors:** Gabriel Macedo Costa Guimarães, Christiane Costa-Pereira, Renan da Silva Faustino, Lilian Santos Alves, Fabiana Rabe Carvalho, Thalia Medeiros, Joaquim Pedro Brito-de-Sousa, Laurence Rodrigues Amaral, Vanessa Peruhype-Magalhães, Ana Carolina Campi-Azevedo, Andréa Teixeira-Carvalho, Andrea Alice Silva, Olindo Assis Martins-Filho

**Affiliations:** 1 Universidade Federal Fluminense, Faculdade de Medicina, Laboratório Multiusuário de Apoio à Pesquisa em Nefrologia e Ciências Médicas, Rio de Janeiro, Brazil; 2 Instituto René Rachou, Fundação Oswaldo Cruz (FIOCRUZ-Minas), Grupo Integrado de Pesquisas em Biomarcadores, Belo Horizonte, Brazil; 3 Departamento de Patologia, Faculdade de Medicina, Universidade Federal Fluminense, Niterói, Rio de Janeiro, Brazil; 4 Laboratório de Bioinformática e Análises Moleculares, Universidade Federal de Uberlândia, Uberlândia, Brazil; 5 Instituto de Investigação em Doenças Infecciosas e Crônicas das Mucosas e Pele (INCT Mucosa e Pele), Belo Horizonte, Brazil

**Keywords:** COVID-19, cytokines, disease outcome, immune mediators, immune response

## Abstract

**Introduction:**

The present study is an observational descriptive follow-up investigation designed to characterize the profile of serum immune mediators in COVID-19 patients further categorized according to disease outcome.

**Methods:**

A total of 92 COVID-19 patients were enrolled in a timeline kinetics, starting at hospital admission (Day 0) throughout consecutive timepoint intervals (Day 3–7, Day 8–14 and Day 15–40). Immune mediators (chemokines, cytokines and growth factors) were quantified by a high-throughput multiplex assay and compared with a pre-pandemic healthy control group (HC).

**Results:**

Data demonstrated that COVID-19 exhibited a classical immune mediator storm, with prominent increase of chemokines and pro-inflammatory cytokines. Longitudinal follow-up revealed that the “death” outcome was associated with a persistent increase of immune mediators across all timepoints, with higher imbalance at Day 8–14. Conversely, the “discharge” outcome evolved with a balanced temporal profile with progressive waning of pro-inflammatory cytokines. Integrative network architectures uncovered that the “death” outcome exhibited a selective high-density chemokine cluster, contrasting with the balanced pattern described for “discharge” subgroups. A set of serum immune mediators (CXCL8, CCL2, CXCL10, IL-6, and IFN-γ) emerged as relevant predictors of disease outcome (AUC ≥ 0.8). Decision tree stepwise algorithms pointed out the hierarchical power (accuracy = 83%) of IL-6, CCL2, and CXCL8 to sort out patients according to disease outcome.

**Conclusions:**

Overall, these findings support clinical applicability of measuring serum immune mediators as complementary prognostic biomarkers for early classification and prediction of disease outcome in COVID-19 patients.

## Introduction

The Severe Acute Respiratory Syndrome Coronavirus 2 (SARS-CoV-2), which causes coronavirus disease 2019 (COVID-19), has triggered a major global health crisis due to its rapid global spread, high transmissibility and potential for progression to severe disease. Although many individuals remain asymptomatic or exhibit moderate illness, some may progress to respiratory failure accompanied by systemic hyperinflammation, frequently requiring intensive care and presenting a higher risk for poor prognosis (1, 2). In COVID-19, progression toward severe complications associated with increased mortality risk is frequently accompanied by dysregulated immune responses characterized by a cytokine storm, condition defined by elevated circulating levels of pro-inflammatory mediators, including chemokines (e.g., CXCL8, CCL2, and CXCL10) and cytokines (e.g., IL-1β, IL-6, IL-10, TNF-α, and IFN-γ), which contribute to leukocyte activation, endothelial dysfunction, and tissue injury (3–6). Therefore, the disruption of regulatory mechanisms promotes uncontrolled inflammation, which is frequently linked to poor prognosis (7, 8).

Multidimensional analytical strategies are required to elucidate the temporal evolution and systemic interplay of soluble immune mediators in COVID-19. A combination of timeline kinetics, signature profiling, integrative networks and decision-tree algorithms provide a comprehensive framework for investigating coordinated immune responses and identifying prognostic biomarkers. Therefore, the present study was designed to explore the timeline and network interplay of immune circulating mediators in COVID-19 patients further categorized according to disease outcome. By profiling coordinated immune signatures across timeline kinetics, this investigation sought to characterize the immunological landscape of COVID-19 and reinforce the clinical applicability of serum immune mediators as complementary tools for early classification and prediction of disease outcomes.

## Population, materials, and methods

### Study population, design, and methods

This is an observational descriptive follow-up investigation conducted during the first wave of the COVID-19 pandemic in Brazil, from April 1^st^ to August 31^st^ 2020, during the circulation of SARS-CoV-2 B.1.1.28 and B.1.1.33 strains. A total of 92 hospitalized patients with RT-qPCR-confirmed SARS-CoV-2 infection (COVID-19) were enrolled at the Hospital Universitário Antonio Pedro (HUAP-UFF/EBSERH), which is a tertiary-to-quaternary referral center affiliated to the Universidade Federal Fluminense (UFF), located in Niterói, Rio de Janeiro, Brazil.

Demographic and clinical data of COVID-19 patients were obtained at enrollment from medical records, including symptoms, comorbidities, complications during hospitalization (Intensive care unit requirement and progression to critical cases) and the length of hospitalization. The COVID-19 group comprised 52 males and 40 females, aged 18 to 93 years (mean age: 59 ± 18 years), and was further categorized according to disease outcome into two subgroups: “discharge” (n = 51) and “death” (n = 41). All patients included in the study were hospitalized, and subgroup classification was based on in-hospital clinical outcome, with the “discharge” subgroup comprising patients who clinically recovered and were discharged alive from the hospital, and the “death” subgroup comprising patients who died during hospitalization. This cohort represents a subset of hospitalized COVID-19 patients with serum samples available for longitudinal immune mediator analyses and should not be interpreted as reflecting overall hospital admission mortality during the study period. A reference group of healthy individuals collected during the pre-pandemic period between February and April 2018 (HC, n = 50), matched by age and sex, was included as a reference control for comparative analysis of serum immune mediators. The HC group comprised 25 males and 25 females, aged from 19 to 80 years (median = 64 years), and all participants presented a minimum interval of at least 30 days between sample collection and any vaccination scheme. This control group was selected as a non-probabilistic convenience sampling from a biorepository maintained at *Grupo Integrado de Pesquisas em Biomarcadores, Instituto René Rachou*, Fundação Oswaldo Cruz (FIOCRUZ-Minas), Belo Horizonte, Brazil.

**Figure 1:**
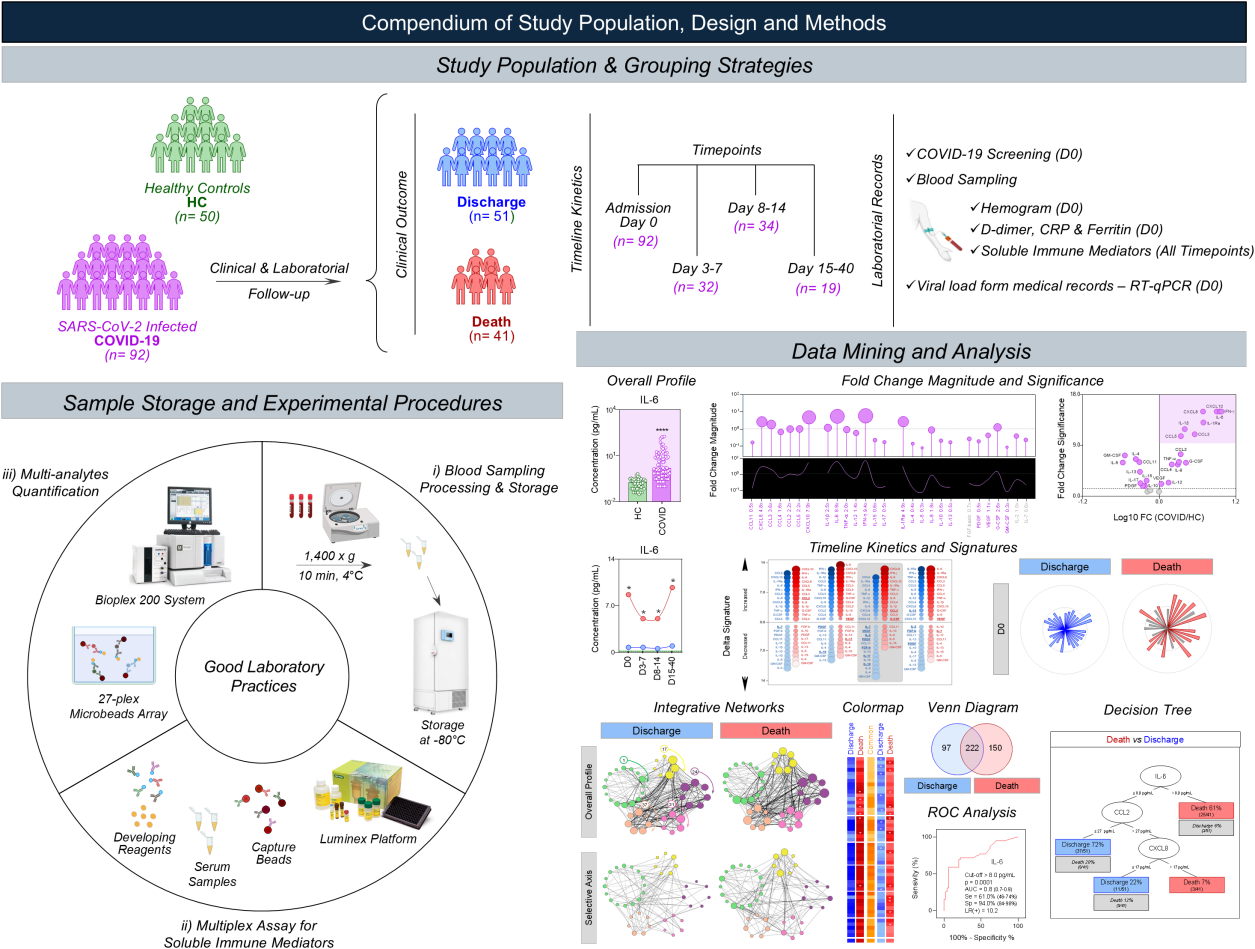
Compendium of study population, design, and methods. This is an observational descriptive follow-up investigation conducted during the first wave of COVID-19 pandemic in Brazil, designed to characterize the serum immune mediator profile in COVID-19 patients according to disease outcomes. As a control group, pre-pandemic healthy subjects (green, HC, n = 50) with no prior exposure were included to establish baseline immune profiles. A total of 92 hospitalized individuals with confirmed SARS-CoV-2 infection (purple, COVID-19, n = 92) were enrolled and further categorized according to disease outcome into two subgroups: those who recovered and were discharged (blue, Discharge, n = 51) and those who died during hospitalization (red, Death, n = 41). Peripheral blood samples were collected from COVID-19 patients at hospital admission (Day 0) and at consecutive timepoint intervals, referred as: Day 3–7, Day 8–14 and Day 15–40. Blood samples were obtained by venipuncture using vacuum system in tubes without anticoagulant as well as in tubes containing EDTA. Serum aliquots from all timepoints were stored at -80 °C until processing for quantification of immune mediators (chemokines, cytokines, and growth factors). The quantification of serum immune mediators was performed using a high-throughput multiplex assay, following the manufacturer instructions. Laboratory records including hematological/biochemical parameters were performed only at hospital admission. Nasopharyngeal viral load data were obtained from archives of medical records. A combination of analytical strategies was employed, including overall profile, fold change magnitude and significance, timeline kinetics, signatures, integrative networks, colormaps, ROC analysis, and decision tree. The figure summarized all steps of the work as the study population & grouping strategies, clinical & laboratorial follow-up, timeline kinetics, sample storage, experimental procedures, and data mining.

Peripheral blood samples were collected from COVID-19 patients at hospital admission (Day 0) and at consecutive time-point intervals, referred to as: Day 3-7, Day 8–14 and Day 15-40. The definition of these temporal windows was based on a real-life hospital setting during the first pandemic wave and aimed to capture early, intermediate, and late phases of disease progression rather than fixed daily intervals. Sample collection beyond admission depended on patient availability, clinical stability, and hospitalization dynamics. Blood samples were obtained by venipuncture using vacuum system in tubes without anticoagulant as well as in tubes containing EDTA for quantification of immune mediators (chemokines, cytokines, and growth factors) and analyses of hematological/biochemical parameters. Serum aliquots from all timepoints were stored at -80 °C until processing for quantification of immune mediators. All pre-analytical steps were carried out according to standardized operating procedures and good laboratory practices. A compendium of study population, design and methods is presented in **Figure 1**.

This study was approved by the Research Ethics Committee of Universidade Federal Fluminense (CAAE: 30623520.5.0000.5243) and by the Ethics Committee of Instituto René Rachou/FIOCRUZ-MG (CAAE: 82357718.5.0000.5091). All participants enrolled in this investigation signed an informed consent form in accordance with the Declaration of Helsinki and Resolution 466/2012 of the Brazilian National Health Council for research involving human subjects.

### Laboratory records – hematological/biochemical parameters

Hematological parameters were obtained from an automated hematological analyzer (Sysmex CA-1500^®^ System; Sysmex America Inc., IL, USA). Biochemical parameters (ferritin and C-reactive protein) were measured using commercially available kits for automated platforms (Coulter LH 750^®^; Beckman Coulter, CA, USA and Dimension RxL Max^®^; Siemens, DE, USA). All routine laboratory tests were performed at the Clinical Pathology Unit (UACAP/HUAP-UFF/EBSERH). Laboratory records comprise hemoglobin, hematocrit, red cell distribution width (RDW), white blood cells (WBC), red blood cells (RBC), platelets, lymphocytes, neutrophils, band cells, monocytes, neutrophil-to-lymphocyte ratio (NLR), monocyte-to-lymphocyte ratio (MLR), D-dimer, C-reactive protein (CRP) and ferritin.

### Viral load records

Viral load data were obtained from archives of medical records. The SARS-CoV-2 viral load was quantified in nasopharyngeal swabs by RT-qPCR assay at the Multiuser Laboratory for Research Support in Nephrology and Medical Sciences (LAMAP-UFF), certified for SARS-CoV-2 testing by the Central Public Health Laboratory Noel Nutels (LACEN-RJ). Briefly, nasopharyngeal swabs were collected from patients upon hospital admission and total viral RNA was extracted using the QIAamp Viral RNA Mini Kit, following the manufacturer instructions (QIAGEN, Hilden, Germany). Amplification and detection of SARS-CoV-2 target genes were carried out using the 2019-nCoV RUO Kit (Catalog #10006770, Integrated DNA Technologies, IDT, USA), in combination with the GoTaq^®^ Probe 1-Step RT-qPCR System (Catalog #A6121, Promega Corporation, USA). Reactions were run on the 7500 Real-Time PCR System (Applied Biosystems, Thermo Fisher Scientific, USA). Cycle threshold (Ct) values below 38 for N1 and N2 targets, and below 35 for the internal control (human RNase P), were considered positive in accordance with CDC (USA) recommendations. The results were expressed as log10 copies/mL.

### Quantification of serum immune mediators

The quantification of serum immune mediators was performed using a high-throughput multiplex assay (Bio-Plex Pro™ Human Cytokine 27-plex Panel, Bio-Rad Laboratories, CA, USA), following the manufacturer instructions. The concentrations of chemokines (CCL11, CXCL8, CCL3, CCL4, CCL2, CCL5, CXCL10), pro-inflammatory cytokines (IL-1β, IL-6, TNF-α, IL-12, IFN-γ, IL-15, IL-17), regulatory cytokines (IL-1Ra, IL-4, IL-5, IL-9, IL-10, IL-13) and growth factors (FGF-basic, PDGF, VEGF, G-CSF, GM-CSF, IL-2, IL-7) were determined using the Bio-Plex^®^ 200 system (Bio-Rad) and expressed as picograms per milliliter (pg/mL).

### Data mining and analysis

The statistical analyses were performed using a combination of software platforms to ensure robust and comprehensive data interpretation. Descriptive statistical analyses were performed, and the data normality test was assessed by the Shapiro-Wilk test using the Prism 8.0 software (GraphPad software, San Diego, USA). Analyses of serum immune mediator concentrations between groups were carried out by the Mann–Whitney U test at each timepoint (D0, D3-7, D8–14 and D15-40). In all cases, a threshold of p-value<0.05 was considered statistically significant.

Fold-change (FC) analyses were conducted to quantify the magnitude of changes in serum levels of immune mediators across all groups. FC values were calculated as the ratio between the median values of each immune mediator in the reported group divided by the median values reported for the counterpart comparative group (COVID-19 vs. HC; Discharge vs. HC; Death vs. HC; and Death vs. Discharge). The magnitude of changes in the serum levels of immune mediators was determined considering: decrease (<1x) and increase (>1x) levels relative to the ratio between the median values of each group. Orbital graphs were generated using Microsoft Excel version 2012. FC values were computed as log10 ratios between group medians and visualized using colormap matrices with gradient scales, allowing the identification of mediators with distinct modulation patterns across clinical groups and timepoints.

Correlation analysis (Pearson and Spearman correlation tests) was used to construct integrative networks. Only moderate and strong (“r” scores > |0.67|) significant correlations (p<0.05) were considered. The Cytoscape software (available at https://cytoscape.org) was used to build circular layouts to display five biomarker clusters, comprising 43 nodes representing: laboratory records (LRec, 1-16), chemokines (C, 17-23), pro-inflammatory cytokines (Pro, 24-30), regulatory cytokines (Reg, 31-36), and growth factors (GF, 37-43). Venn diagram analysis (available at http://bioinformatics.psb.ugent.be/webtools/Venn/) was carried out to identify the common and selective correlation axes observed in “discharge” and “death” subgroups.

Single-parameter and combined stepwise analyses of serum immune mediators were evaluated to identify the most accurate approach for classifying COVID-19 patients according to disease outcome. Receiver Operating Characteristic (ROC) curve analyses were constructed using GraphPad Prism 8.0 software (San Diego, CA, USA) to estimate the performance indices of single-step analysis of serum immune mediators. Performance metrics (area under the ROC curve = AUC; Sensitivity = Se; Specificity = Sp, and optimal cutoff values = Cut-off) were calculated to assess the ability of single immune mediators to classify “discharge” and “death” subgroups at hospital admission. In addition to the single-parameter analysis, a stepwise decision tree algorithm was constructed to assess the accuracy of serum immune mediators, preselected in the ROC analysis, for classifying COVID-19 patients according to disease outcome. Leave-one-out cross-validation (LOOCV) was employed as an additional performance index to generalize the findings of the statistical model to an independent dataset. These analyses were performed using WEKA software, version 3.6.11 (University of Waikato, New Zealand, Australia).

## Results

### Overall profile of COVID-19 patients at hospital admission

At admission, demographic and clinical data analysis demonstrated that the most frequent symptoms in the COVID group included fever, cough, dyspnea and fatigue, and cardiovascular disease was the most common comorbidity, as shown in **Table 1**. When patients were stratified by disease outcome, those who progressed to death were older than those discharged. In addition, among the symptoms reported at admission, myalgia was less frequent in the “death” subgroup. Regarding comorbidities and clinical complications, acute kidney injury, diabetes mellitus and in-hospital complications were more frequent in the “death” subgroup. Moreover, the length of hospitalization was longer among patients who progressed to death. Furthermore, laboratory records including hematological and biochemical parameters obtained at admission showed alterations indicative of an inflammatory response in COVID-19 patients, with more pronounced changes observed in the “death” subgroup as compared to “discharge” subgroup. Comparative laboratory data analysis according to disease outcome are summarized in **Supplementary Table S1**.

**Table 1:** Demographic and clinical parameters of COVID-19 patients at hospital admission classified according to disease outcome.

Parameters	COVID (n=92)	COVID subgroups		
		Discharge (n=51)	Death (n=41)	P-Value
Age, years (mean ± SD)	59 ± 18	**56 ± 18**	**64 ± 16**	**0.02**
Male gender, n (%)	52 (56)	26 (50)	26 (63)	0.2
Symptoms at admission, n (%)				
Fever	62 (67)	38 (74)	24 (58)	0.1
Cough	58 (63)	32 (63)	26 (63)	0.9
Sore throat	5 (5)	4 (7)	1 (2)	0.3
Headache	16 (17)	9 (18)	7 (17)	0.9
Fatigue	34 (37)	23 (46)	11 (27)	0.08
Myalgia	14 (15)	**12 (23)**	**2 (5)**	**0.01**
Anosmia/ageusia	15 (16)	11 (22)	4 (10)	0.1
Diarrhea	14 (15)	8 (15)	6 (15)	0.9
Dyspnea	52 (56)	27 (53)	25 (60)	0.5
Hypoxia (O_2_ sat. < 95%)	39 (42)	21 (41)	18 (44)	0.6
Comorbidities, n (%)				
Cancer	31 (34)	14 (28)	17 (41)	0.1
CVD	58 (63)	32 (63)	26 (63)	0.9
CKD	12 (13)	6 (11)	6 (15)	0.7
AKI	40 (44)	**12 (24)**	**28 (68)**	**0.0001**
Diabetes	29 (31)	**11 (22)**	**18 (36)**	**0.02**
Obesity	18 (19)	9 (18)	9 (21)	0.6
Immunosuppression	20 (22)	11 (22)	9 (21)	0.9
Complications during hospitalization, n (%)				
ICU Requirement	56 (60)	**21 (41)**	**35 (86)**	**0.0001**
Critical Cases	44 (48)	**9 (17)**	**35 (86)**	**0.0001**
Length of hospitalization (days, mean ± SD)	25 ± 21	**30 ± 25**	**19 ± 13**	**0.01**

Data are presented as n (%) or mean ± SD. CKD, chronic kidney disease; CVD, cardiovascular disease; AKI, acute kidney injury; ICU, intensive care unit. Critical cases were defined as the requirement of invasive mechanical ventilation and hemodynamic instability. Comparative analysis between Death vs Discharge was performed by t-student, Mann-Whitney test or Fisher’s exact test. Significant differences at p<0.05 are underscored by bold format.

### Overall profile of serum immune mediators in COVID-19 patients at hospital admission

**Figure 2:**
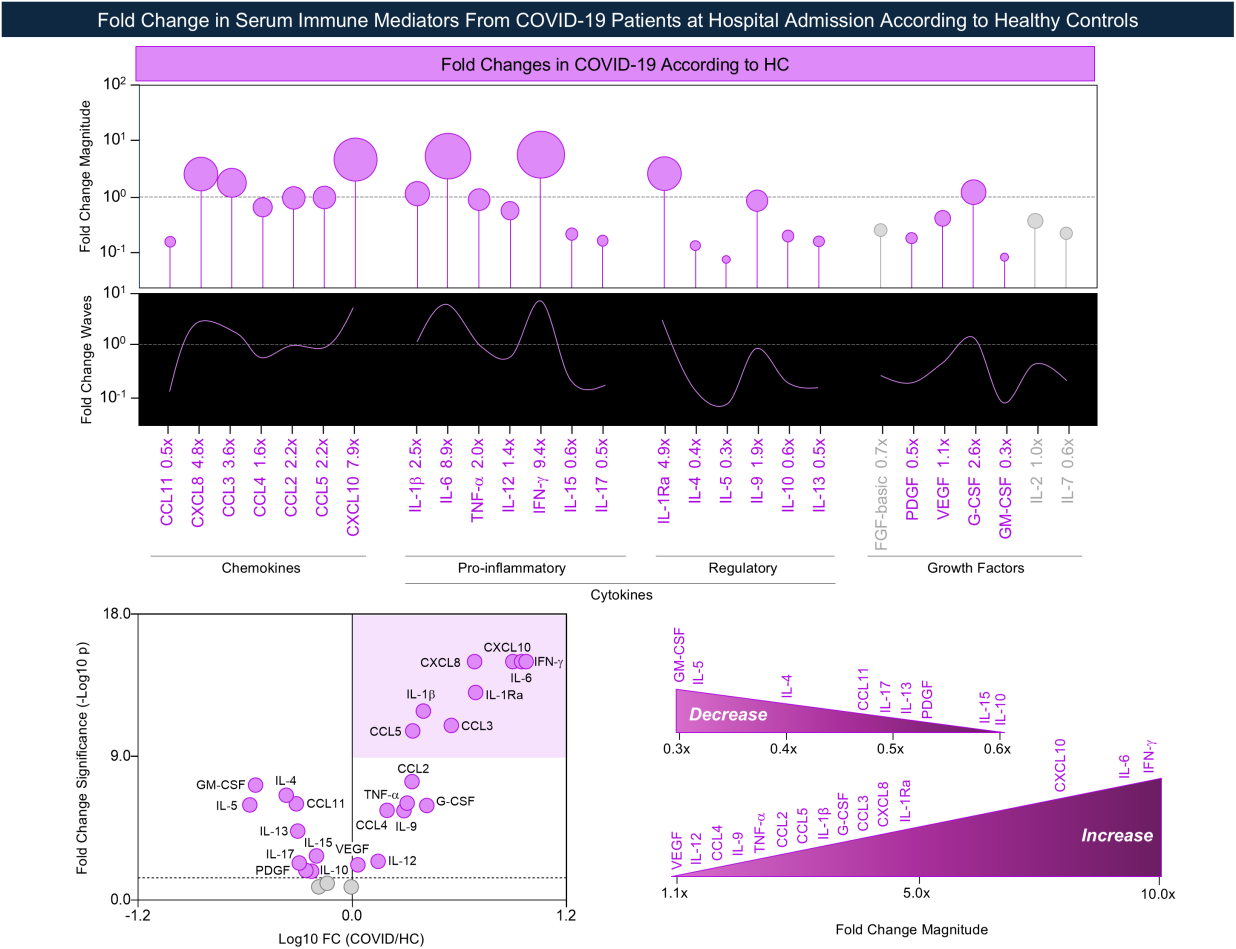
Fold change in serum immune mediators from COVID-19 patients at hospital admission according to healthy controls. The panoramic profile of changes on chemokines, pro-inflammatory cytokines, regulatory cytokines, and growth factors was assessed in serum samples from COVID-19 patients (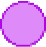, COVID-19, n = 92) at hospital admission according to Healthy Controls (HC, n = 50). Measurements of serum soluble mediators were carried out by Luminex Bio-plex platform as described in Material and methods section. The fold change value was calculated for each immune mediator as the ratio between the median concentration of COVID group according to the median values reported for HC. The results are shown in lollipop charts representing the median fold changes in serum immune mediators observed for COVID group at hospital admission according to HC. Significant fold change values at p <0.05, referred as decreased (FC < 1.0) or increased levels (FC > 1.0), were underscored by pink symbols. Non-significant FC values are represented by gray symbols. The volcano-like plot was constructed based on fold change magnitude (Log10 Fold Change) versus significance (-Log10 p-value) to identify the set of serum immune mediators with more prominent changes in COVID group as compared to HC, underscored by pink background.

Fold-change analysis demonstrated that the COVID group presented increased FC for 15 out of 27 (55%) immune mediators, ranging from 9.4x to 1.1x, with ranked profile as follows: IFN-γ (9.4x), IL-6 (8.9x), CXCL10 (7.9x), IL-1Ra (4.9x), CXCL8 (4.8x), CCL3 (3.6x), G-CSF (2.6x), IL-1β (2.5x), CCL5 (2.2x), CCL2 (2.2x), TNF-α (2.0x), IL-9 (1.9x), CCL4 (1.6x), IL-12 (1.4x), and VEGF (1.1x). Conversely, the COVID group exhibited decreased FC magnitude for 9 out of 27 (33%) immune mediators, ranging from 0.3x to 0.6x, with ranked profile as follows: GM-CSF (0.3x), IL-5 (0.3x), IL-4 (0.4x), CCL11 (0.5x), IL-17 (0.5x), IL-13 (0.5x), PDGF (0.5x), IL-15 (0.6x), and IL-10 (0.6x). No significant differences were observed in the serum levels of FGF-basic, IL-2, and IL-7. Of note, volcano-like plot analysis pointed out a set of eight immune mediators (CXCL8, CCL3, CCL5, CXCL10, IL-1β, IL-6, IFN-γ, and IL-1Ra) with higher significance of change in COVID group as compared to HC (**Figure 2**). These findings are supported by the distribution of serum immune mediator levels shown in **Supplementary Figure S1**, which provides an additional overview of the overall profile of COVID-19 patients compared to healthy controls.

**Figure 3:**
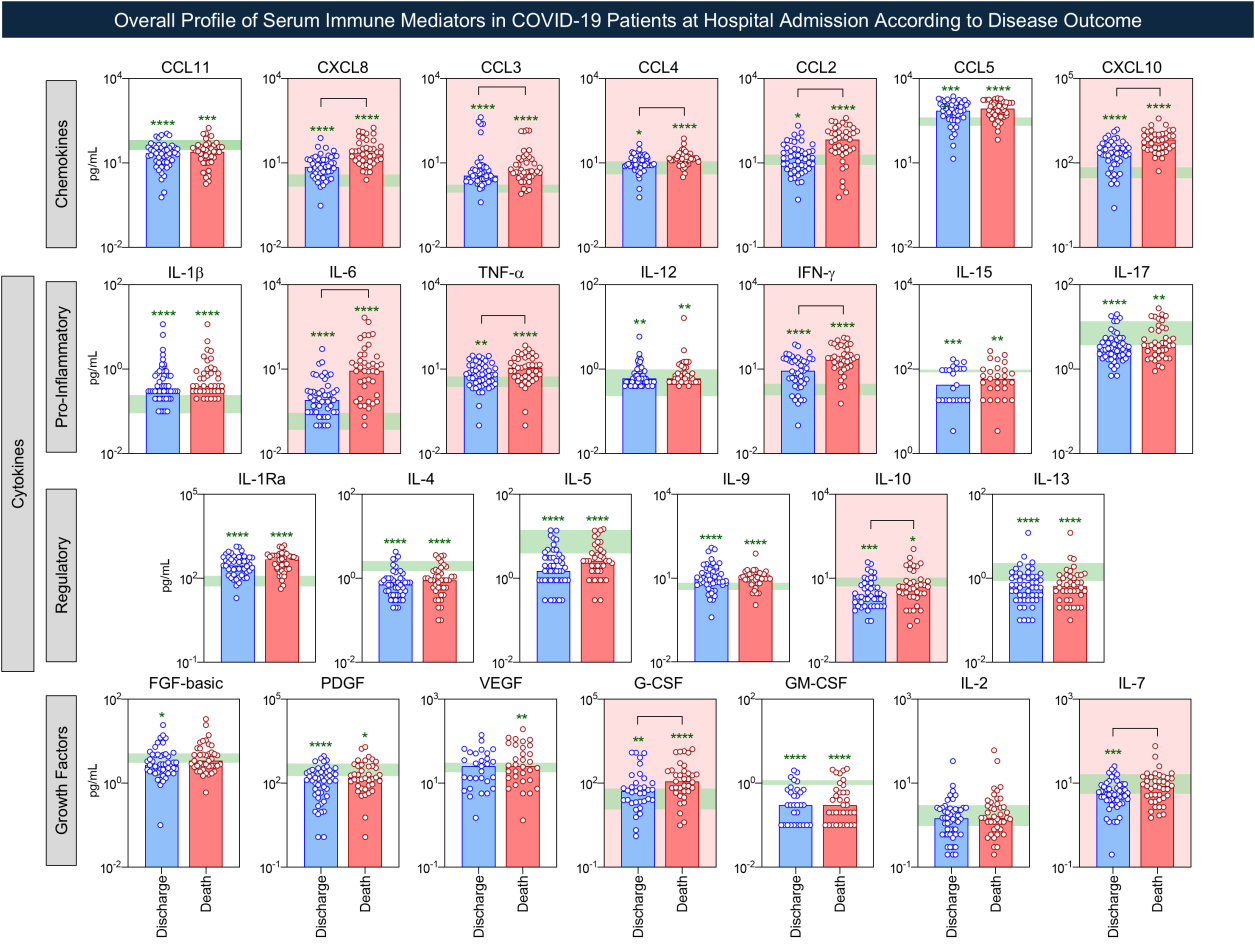
Overall profile of serum immune mediators in COVID-19 patients according to disease outcome. The overall profile of chemokines, pro-inflammatory cytokines, regulatory cytokines, and growth factors was evaluated in serum samples from COVID-19 patients at hospital admission, further categorized according to disease outcome [Discharge (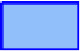, Discharge, n = 51) and Death (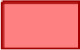, Death, n = 41)] as compared to Healthy Controls (green-shaded zone representing the 25^th^-75^th^ interquartile range, n = 50). Measurements of serum mediators were carried out by Luminex Bio-plex platform as described in Material and methods section. The results are presented as the scattering distribution of individual values over bars underscoring the median values of serum immune mediators. Comparative analysis between Discharge and Death subgroups and HC reference values was performed by the Mann-Whitney test. Significant differences for comparative analysis with HC are represented by *, **, *** or **** to denote the p-values <0.05, <0.01, <0.001 or <0.0001, respectively. Significant differences between Discharge vs Death are identified by connecting lines. The red background was used to underscore the serum immune mediators with increased levels in patients evolving to Death as compared to Discharge outcome.

Further, to identify the immunological profile associated with disease outcome, serum immune mediators were compared between groups. The results are shown in **Figure 3**. Data analysis demonstrated that both COVID subgroups exhibited elevated levels of almost all serum immune mediators, except for IL-2, as compared to HC. Of note, the “death” subgroup presented higher levels of CXCL8, CCL3, CCL4, CCL2, CXCL10, IL-6, TNF-α, IFN-γ, IL-10, G-CSF, and IL-7 as compared to “discharge” subgroup, exemplifying an exacerbated storm of immune mediators (**Figure 3**).

**Figure 4:**
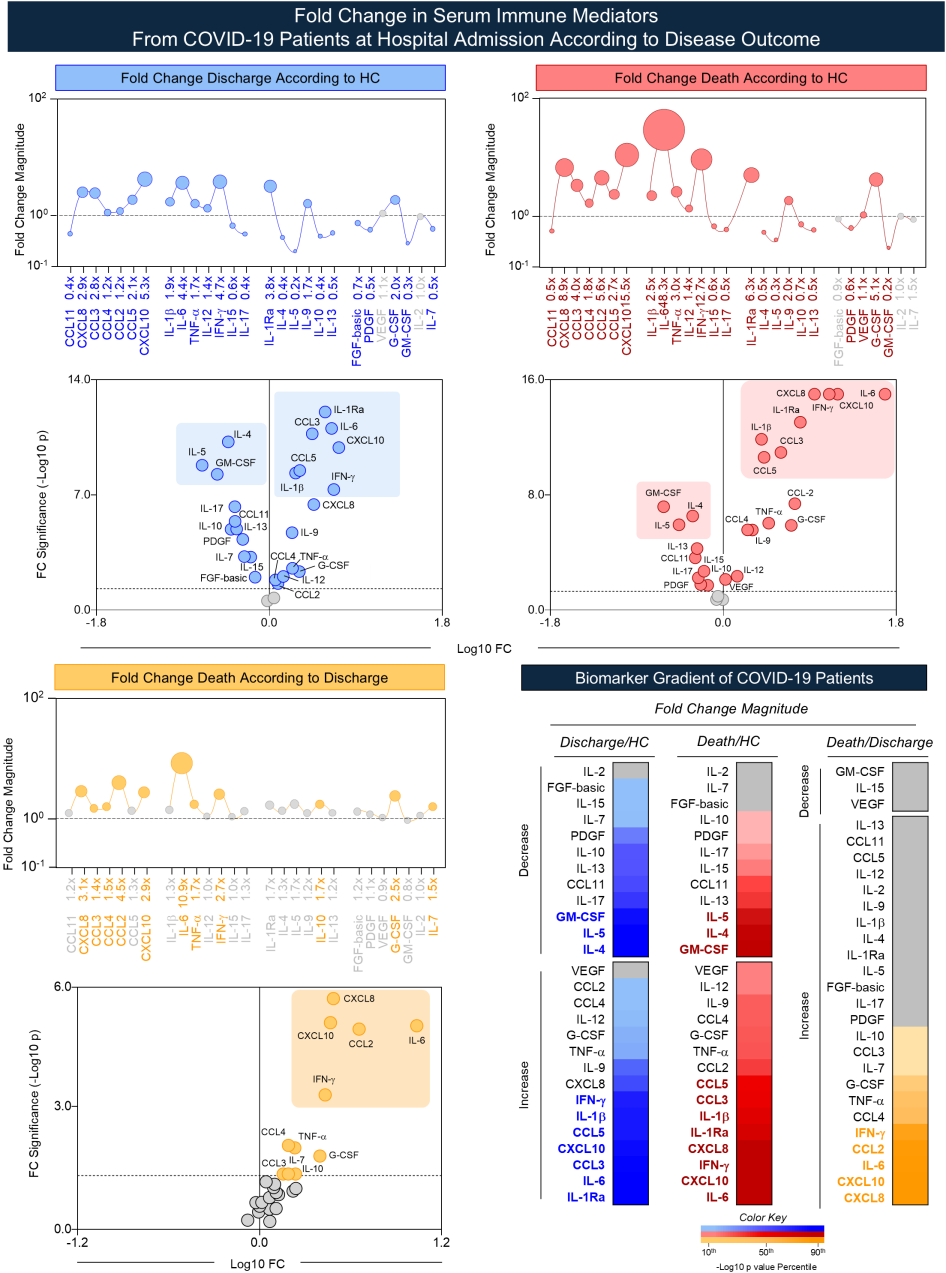
Fold change in serum immune mediators from COVID-19 patients at hospital admission according to disease outcome. The panoramic profile of changes on chemokines, pro-inflammatory cytokines, regulatory cytokines, and growth factors was assessed in serum samples from COVID-19 patients, further categorized according to disease outcome [Discharge (n = 51), and Death (n = 41)] and compared to HC (n = 50). Measurements of serum mediators were carried out by Luminex Bio-plex platform as described in Material and methods section. The fold change value was calculated for each immune mediator as the ratio between the median concentration of COVID subgroups according to the median values reported for HC, referred as: Discharge/HC (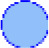) and Death/HC (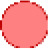) as well as Death/Discharge (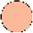). The results are shown in orbital plots over lines representing the median fold changes in serum immune mediators observed for Discharge vs HC, Death vs HC, and Death vs Discharge. Significant fold change values [decreased (FC < 1.0) or increased levels (FC > 1.0)] were underscored by colored symbols. Non-significant FC values are represented by gray symbols. The volcano-like plots were constructed based on fold change magnitude (Log10 Fold Change) vs significance (-Log10 p-value) to identify the set of serum immune mediators with more prominent changes in COVID subgroups. A color key was applied to represent the biomarker gradient of log10 FC.

Comparative data analysis further demonstrated that the “discharge” group presented increased FC for 14 out of 27 (52%) immune mediators according to HC, ranging from 5.3x to 1.2x. Volcano-like plot analysis identified a set of eight immune mediators [CXCL10 (5.3x), IFN-γ (4.7x), IL-6 (4.4x), IL-1Ra (3.8x), CXCL8 (2.9x), CCL3 (2.8x), CCL5 (2.1x), and IL-1β (1.9x)] with higher increase significance in “discharge” subgroup as compared to HC, as shown in **Figure 4**. On the other hand, the same group also showed decreased FC for 11 out of 27 (40%) immune mediators according to HC, ranging from 0.2x to 0.7x. Volcano-like plot analysis identified a set of three immune mediators [IL-5 (0.2x), GM-CSF (0.3x), and IL-4 (0.4x)] with higher decrease significance in “discharge” subgroup as compared to HC. The analysis of “death” subgroup showed increased fold changes for 15 out of 27 (56%) immune mediators according to HC, ranging from 48.3x to 1.1x, with a set of eight immune mediators [IL-6 (48.3x), CXCL10 (15.5x), IFN-γ (12.7x), CXCL8 (8.9x), IL-1Ra (6.3x), CCL3 (4.0x), CCL5 (2.7x), and IL-1β (2.5x)] presenting higher increase significance as compared to HC. Conversely, decreased FC were observed for 9 out of 27 (33%) immune mediators in “death” subgroup according to HC, ranging from 0.2x to 0.7x. Volcano-like plot analysis demonstrated a set of three immune mediators [GM-CSF (0.2x), IL-5 (0.3x), and IL-4 (0.5x)] with higher significance of decrease in “death” subgroup as compared to HC. Further comparative analysis between the “death” and “discharge” subgroups demonstrated increased FC magnitude for 9 out of 27 (33%) immune mediators, ranging from 10.9x to 1.5x. Volcano-like plot analysis pointed out a set of five immune mediators [IL-6 (10.9x), CCL2 (4.5x), CXCL8 (3.1x), CXCL10 (2.9x), and IFN-γ (2.7x)] with higher increase significance in “death” as compared to “discharge” subgroup. Colormap constructs were assembled to illustrate the overall fold change magnitude with higher significance (-log_10_ p values) in “discharge” and “death” subgroups according to HC (Discharge/HC; Death/HC) as well as “death” subgroup as compared to “discharge” subgroup (Death/Discharge) (**Figure 4**).

**Figure 5:**
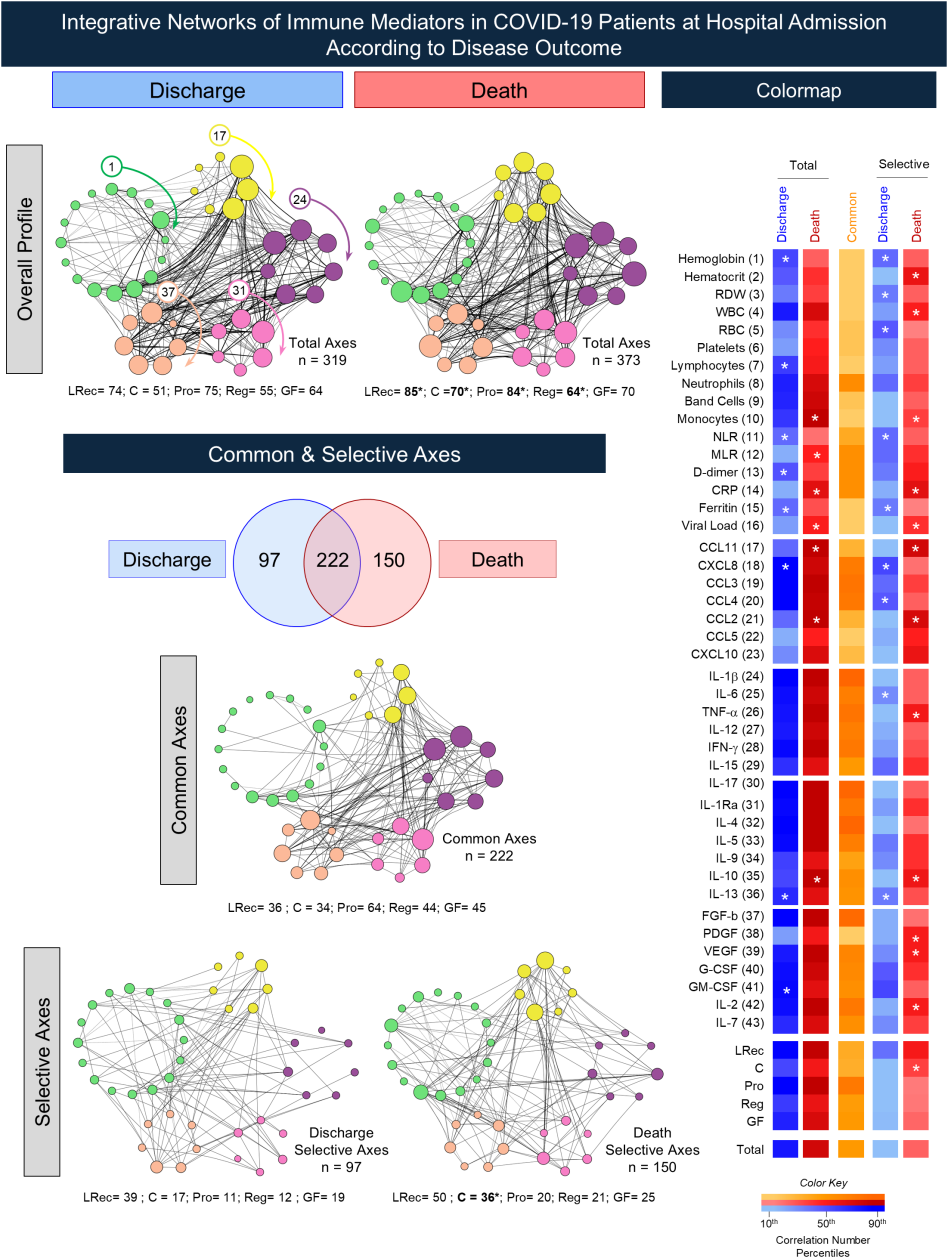
Integrative networks of immune mediators in COVID-19 patients according to disease outcome. Integrative networks were constructed for Discharge (n = 51) and Death (n = 41) subgroups at the hospital admission (D0). based on Pearson and Spearman rank correlation tests. Circular cluster layouts to display five biomarker clusters, comprising 43 nodes representing: laboratory records (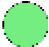
= LRec, 1-16), chemokines (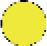
= C, 17-23), pro-inflammatory cytokines (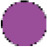
= Pro, 24-30), regulatory cytokines (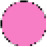
= Reg, 31-36), and growth factors (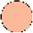
= GF, 37-43). Node sizes are proportional to the number of correlations with continuous and dashed lines indicating positive and negative correlations between nodes, respectively. Venn Diagram analysis was performed to identify common and selective axes between Discharge and Death outcomes. Descriptive analysis of network connectivity including total axes and number of axes by biomarker categories (LRec, C, P, R, and GF) is provided in the figure. The Chi-square test was employed for comparative analysis between Discharge and Death subgroups. Significant differences between Discharge and Death subgroups are represented by * symbol. Colormap construct illustrates the connectivity patterns comprising total, common, and selective axes observed for Discharge and Death subgroups. A color key representing the correlation number gradient is provided in the Figure. Most prominent color gradient observed in Discharge or Death subgroups are underscored by *.

To explore the interplay between laboratory parameters and serum immune mediators in COVID-19 patients according to disease outcome at admission, integrative networks were constructed employing the concept of systems biology and the results are shown in **Figure 5**. Correlation analysis was used to identify moderate and strong significant correlations to assemble integrative networks. Circular layouts, comprising five biomarker clusters (LRec, C, Pro, Reg, and GF) and 43 nodes representing each network attribute. Data analysis demonstrated that, overall, the “death” subgroup exhibited a higher number of connectivity axes (n = 373) as compared to “discharge” (n = 319). The same subgroup exhibited higher numbers of axes within LRec (n = 85), C (n = 70), Pro (n = 84), and Reg clusters (n = 64) (vs. LRec = 74, C = 51, Pro = 75 and Reg = 55). No differences were observed for the GF cluster (Discharge = 64; Death = 70). The analysis of common axes revealed a higher number of shared axes between COVID subgroups (n = 222). However, outcomes were associated with a distinct pattern of selective axes (n = 97 and 150, respectively), with most differences characterized by higher connectivity within the C cluster (Discharge = 11 and Death = 36). Colormap constructs further illustrate these findings, pointing out the most prominent color gradient observed between subgroups (**Figure 5**).

### Timeline kinetics of serum immune mediators in COVID-19 patients according to disease outcome

**Figure 6:**
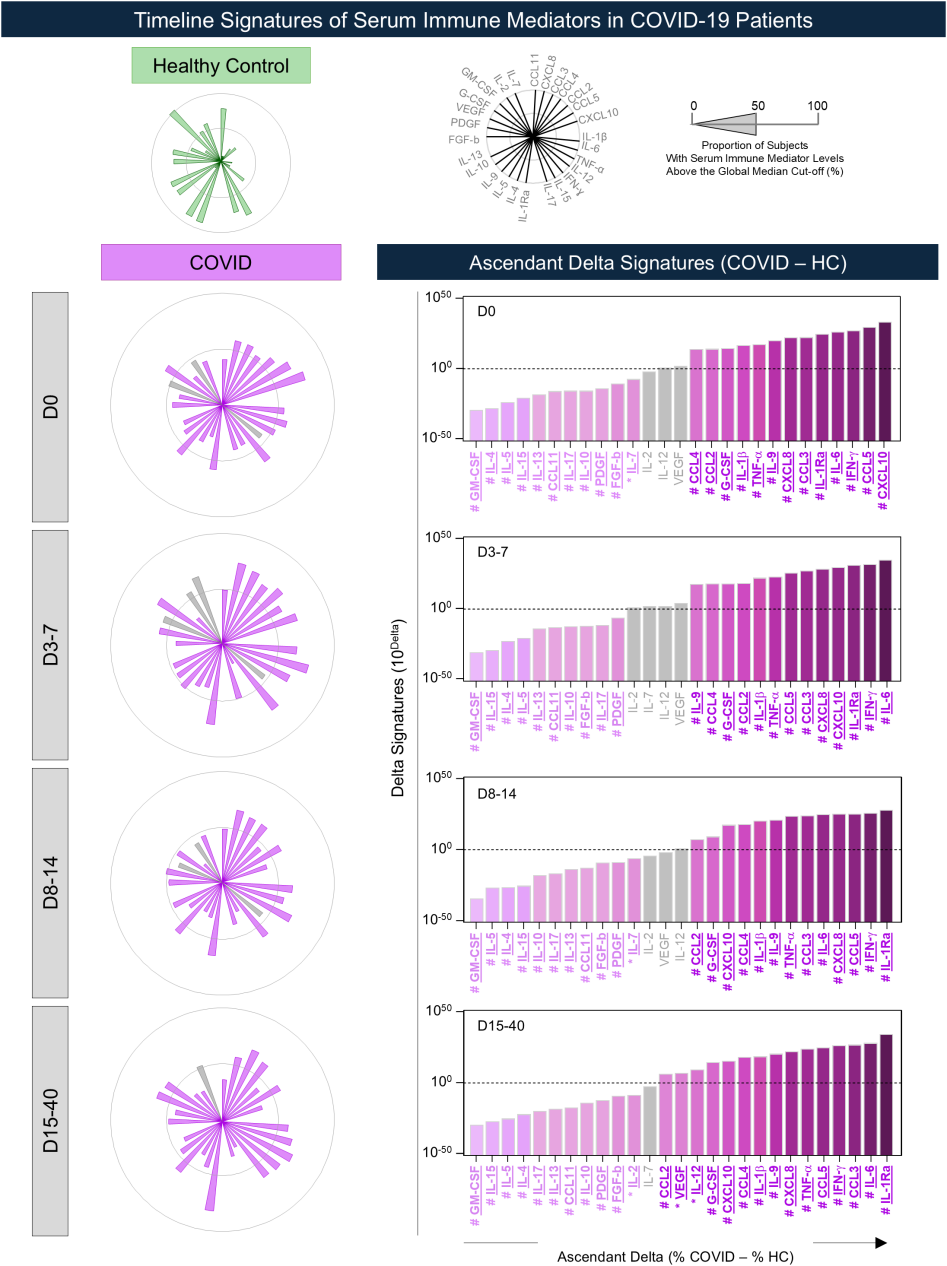
Timeline signatures of serum immune mediators in COVID-19 patients. The overall signatures and ascendant delta of chemokines, pro-inflammatory cytokines, regulatory cytokines, and growth factors were evaluated in serum samples from COVID-19 patients (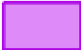, COVID, n = 92) throughout consecutive timepoints following hospital admission (D0), referred as D3-7, D8–14, and D15-40, as compared to Healthy Controls (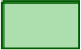, HC, n = 50). Sample availability varied across time windows, with the following number of COVID-19 patients contributing samples: D0 (n = 92), D3-7 (n = 32), D8-14 (n = 34), and D15-40 (n = 19). Measurements of serum mediators were carried out by Luminex Bio-plex platform as described in Material and methods section. Data are shown in radar charts representing the proportion of subjects with serum levels above the global median cut-off (%), calculated for each immune mediator. Comparative analysis amongst COVID-19 timeline subgroups and HC was carried out by Fisher test. Significant differences at p < 0.05 in comparison to HC are underscored by pink bars. Non-significant data are represented by gray bars. Ascendant delta signatures (% COVID – % HC) are presented in color gradient bar charts underscoring significant increase or decrease in serum immune mediators between COVID vs HC along the timeline kinetics. Common and selective serum immune mediators identified along the timeline kinetics are underscored by # and * symbols, respectively.

Sample availability varied over follow-up, with the following number of participants contributing samples at each time window: Day 0 (n = 92), Day 3–7 (n = 32), Day 8–14 (n = 34), and Day 15–40 (n = 19), with corresponding distributions by disease outcome indicated in the figure legends. To further characterize the profile of serum immune mediators along the follow-up, overall signatures were constructed for COVID group throughout consecutive timepoints. The results, expressed as the proportion of subjects with serum levels above the median, are presented in **Figure 6**. Comparative analysis demonstrated that most immune mediators differ in COVID-19 subgroups as compared to HC. The analysis of ascendant delta signature profiles (% COVID - % HC) demonstrated a common increase of CXCL8, CCL3, CCL4, CCL2, CCL5, CXCL10, IL-1β, IL-6, IFN-γ, TNF-α, IL-1Ra, IL-9, and G-CSF throughout the timeline kinetics, with selective increases of IL-12 and VEGF at D15-40. Conversely, a decrease of CCL11, IL-15, IL-17, IL-4, IL-5, IL-10, IL-13, FGF-basic, PDGF, and GM-CSF were observed in COVID subgroups as compared to HC throughout all timepoints, with selective decrease of IL-7 observed at D0 and D8–14 and IL-2 at D15-40 (**Figure 6**).

**Figure 7:**
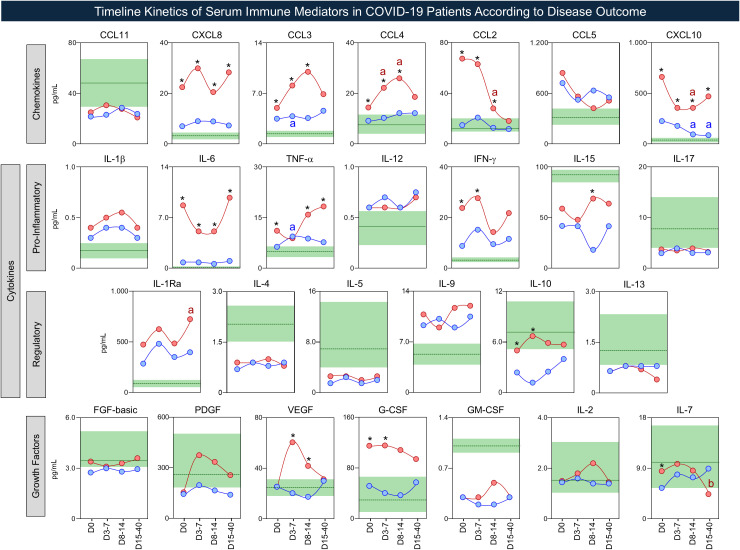
Timeline kinetics of serum immune mediators in COVID-19 patients according to disease outcome. The panoramic timeline kinetics of chemokines, pro-inflammatory cytokines, regulatory cytokines, and growth factors was evaluated in serum samples from COVID-19 patients, further categorized according to disease outcome, referred as: Discharge (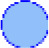, Discharge, n = 51), and Death (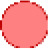, Death, n = 41). Sample availability varied across time windows, with the following number of participants contributing samples: D0 (n = 92; Discharge = 51, Death = 41), D3-7 (n = 32; Discharge = 13, Death = 19), D8-14 (n = 34; Discharge = 21, Death = 13), and D15-40 (n = 19; Discharge = 12, Death = 7). Measurements of serum soluble mediators were carried out by Luminex Bio-plex platform as described in Material and methods section. Intergroup comparisons at each timepoint (Discharge vs Death), as well as intragroup comparative analysis overtime (D0 vs D3–7 vs D8–14 vs D15-40) were performed by the Mann-Whitney test. The results are reported as a line chart expressing the median values observed of serum immune mediators along the timeline kinetics. Significant differences at *p*-value threshold ≤ 0.05 are represented by (*) for intergroup comparisons (Discharge vs Death). Significant intragroup differences are indicated by the letters “a” or “b”, displayed in blue for Discharge subgroup and red for Death subgroup, denoting comparisons relative to early timepoints (D0 and D3-7), respectively. The dashed line and green-shaded zone represent the median value and interquartile range (25^th^–75^th^) of serum immune mediator levels observed in HC (n = 50).

The temporal kinetics of serum immune mediators were further characterized in COVID-19 patients according to disease outcome, as shown in **Figure 7**. Data analysis demonstrated four overall patterns of differences between subgroups along the timeline kinetics, including: i) sustained increase; ii) early increase; iii) early/intermediate rising and iv) transient peak. Based on these patterns, the results pointed out a cluster of three serum immune mediators (CXCL8, CXCL10, and IL-6) with sustained increase in the “death” subgroup. While an early increase was observed for CCL2, IFN-γ, IL-10, and G-CSF, early/intermediate rising was identified for CCL3, and CCL4. A transient peak was observed for TNF-α, IL-15, VEGF, and IL-7 (**Figure 7**).

**Figure 8:**
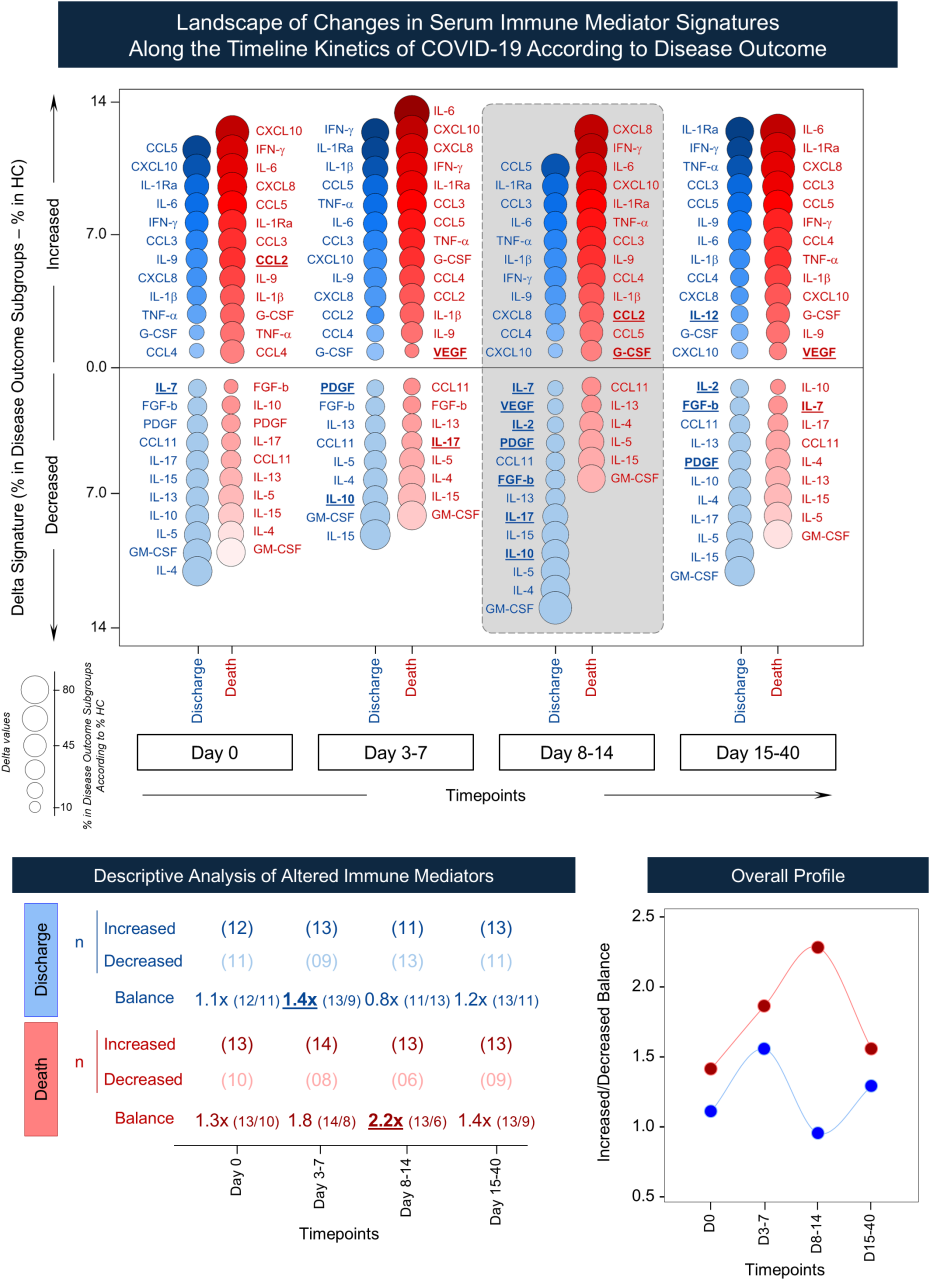
Landscape of changes in serum immune mediator signatures along the timeline kinetics of COVID-19 according to disease outcome. The panoramic profile of chemokines, pro-inflammatory cytokines, regulatory cytokines, and growth factors was evaluated in serum samples of Discharge (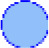, n = 51) and Death (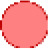, n = 41) subgroups as compared to healthy controls (HC) at consecutive timepoint intervals (D0, D3-7, D8–14, and D15-40). Sample availability varied across time windows, with the following number of participants contributing samples: Day 0 (n = 92; Discharge = 51, Death = 41), Day 3-7 (n = 32; Discharge = 13, Death = 19), Day 8-14 (n = 34; Discharge = 21, Death = 13), and Day 15-40 (n = 19; Discharge = 12, Death = 7). Bubble plots were assembled to display the changes in immune mediators along the timeline kinetics in the Discharge and Death subgroups. Each bubble represents the magnitude of changes in immune mediator signatures, reported as delta signatures calculated as % in Disease Outcome Subgroups – % in HC. Immune mediators with selective increase or decrease between discharge and Death are highlighted across the timeline kinetics by underline format. Serum immune mediators identified descriptive analysis of altered immune mediators (increased/decreased balance) as well as the overall profile observed in Discharge and Death subgroups are presented by line charts.

Furthermore, the landscape of changes in serum immune mediator signatures was investigated in COVID subgroups during the follow-up. The results are presented as delta signatures (% in Disease outcome – % in HC) in **Figure 8**. Data analysis demonstrated a divergent balance between increase and decrease of soluble mediators throughout the timeline kinetics associated with distinct disease outcome. Descriptive analysis of altered immune mediators showed that while progression to discharge exhibited a more balanced temporal profile (balance ~ 1.0x), with a minor peak at D3-7, the “death” outcome was associated with a sustained imbalance (balance > 1.0x) of immune mediators across all timepoints, with pronounced peak at D8–14. The overall profile underscored that major differences between subgroups occurred at D8-14, with an increase of CCL2, and G-CSF in the “death” subgroup, contrasting with the decrease of IL-7, VEGF, IL-2, PDGF, FGF-basic, IL-17, and IL-10 observed in the “discharge” outcome (**Figure 8**). Additional details on the distribution of altered immune mediators at each timepoint are provided in **Supplementary Figure S2**.

### Overall performance of serum immune mediators to categorize COVID-19 patients according to disease outcome

**Figure 9:**
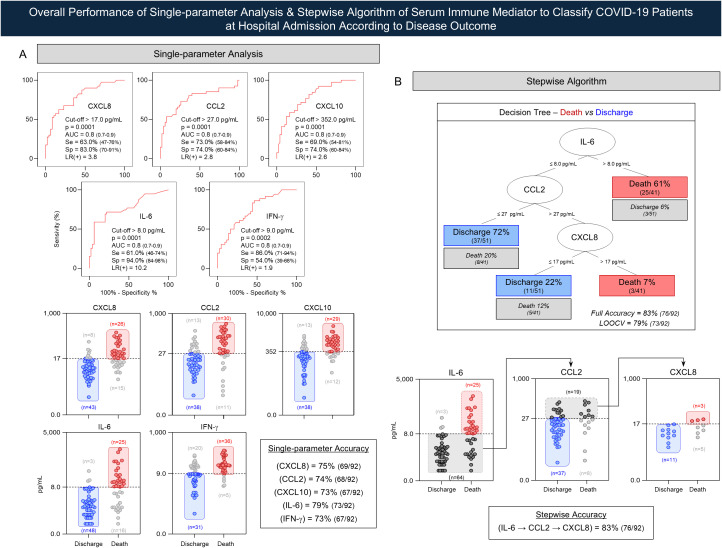
Overall performance of single-parameter analysis & stepwise algorithm of serum immune mediator to classify COVID-19 patients at hospital admission according to disease outcome. **(A)** Receiver Operating Characteristic (ROC) analyses were performed to assess the predictive potential of selected immune mediators (CXCL8, CCL2, CXCL10, IL-6, and IFN-γ) to categorize COVID-19 patients who recovered (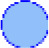, Discharge, n = 51) and those who progressed to death (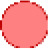, Death, n = 41) at hospital admission. ROC curves are shown with corresponding performance indices including the optimal cut-off values, area under the curve (AUC), sensitivity (Se), specificity (Sp), and likelihood ratio (LR). Scatter plots display the distribution of individual serum concentrations at hospital admission according to disease outcome. The dashed horizontal line represents the selected cut-off value from the ROC curve used to discriminate between Discharge and Death subgroups. Single-parameter analysis of serum immune mediators (CXCL8, CCL2, CXCL10, IL-6, and IFN-γ) was performed based on ROC curve-derived AUC metrics and the accuracy based on true and false classification of Discharge and Death subgroups. Background rectangles underscore correct classifications of patients evolving to Death (red) and Discharge (blue) outcomes. **(B)** Decision tree algorithm of serum immune mediators was constructed to classify Death (red) and Discharge (blue) subgroups based on root (IL-6) and the branch attributes (CCL2 and CXCL8). The full accuracy and the l"eave-one-out cross-validation” (LOOCV) values are provided in the decision tree box. Stepwise scatter plots illustrate the correct classifications of patients evolving to Death (red background) and Discharge (blue background) as well as the transition steps to classify patients evolving to Death and Discharge outcomes. In all cases, gray dots indicate misclassified cases. The single-parameter and stepwise accuracy are provided in the Figure.

ROC curve analysis demonstrated that 5 out of 27 immune mediators (CXCL8, CCL2, CXCL10, IL-6, and IFN-γ) presented useful global accuracy performance (AUC = 0.8) to classify “death” from “discharge” outcome (**Table 2**). These immune mediators were further analyzed employing likelihood ratio scores (LR). Data demonstrated that IL-6 was the parameter with outstanding ability to differentiate groups with LR(+) = 10.2 (**Figure 9**). Stepwise decision-tree algorithm was further employed to estimate the accuracy of immune mediators to classify subgroups. Data pointed out a hierarchical algorithm comprising: IL-6 (cut-off = 8.0 pg/mL) as the root attribute, followed by CCL2 (cut-off = 27 pg/mL) and CXCL8 (cut-off = 17 pg/mL) as branch parameters to correctly classify 94% (48/51) of patients from the “discharge” subgroup and 68% (28/41) from “death” subgroup, reaching an overall accuracy of 83% (76/92). Together, these data support the applicability of measuring the serum levels of IL-6, CCL2, and CXCL8 as complementary prognostic biomarkers for early classification and prediction of disease outcome in COVID-19 patients.

**Table 2:** Performance indices of immune mediators to classify COVID-19 patients at hospital admission according to disease outcome.

Immune Mediators (Cut-off)	Death vs Discharge		
	AUC	Se % (IQR)	Sp % (IQR)
CXCL8 (> 17 pg/mL)	**0.8**	**63 (47-76)**	**83 (70-91)**
CCL2 (> 27 pg/mL)	**0.8**	**73 (58-84)**	**74 (60-84)**
CXCL10 (> 352 pg/mL)	**0.8**	**69 (54-81)**	**74 (60-84)**
IL-6 (> 8.0 pg/mL)	**0.8**	**61 (46-74)**	**94 (84-98)**
IFN-γ (> 9.0 pg/mL)	**0.8**	**86 (71-94)**	**54 (39-67)**
G-CSF (> 66 pg/mL)	0.7	71 (55-84)	66 (49-79)
CCL4 (> 11 pg/mL)	0.7	75 (60-86)	57 (43-70)
IL-10 (> 3.0 pg/mL)	0.6	76 (61-86)	52 (38-66)
CCL3 (> 4.0 pg/mL)	0.6	71 (56-82)	55 (41-68)
IL-7 (> 7.0 pg/mL)	0.6	61 (46-74)	65 (51-76)
TNF-α (> 8.0 pg/mL)	0.6	61 (46-74)	63 (48-75)
IL-4 (> 1.0 pg/mL)	0.6	59 (43-72)	65 (50-77)
IL-1Ra (> 484 pg/mL)	0.6	50 (35-65)	71 (58-82)
IL-15 (> 27 pg/mL)	0.6	78 (59-89)	44 (26-63)
VEGF (> 67 pg/mL)	0.6	28 (16-45)	93 (78-99)
IL-17 (> 4.0 pg/mL)	0.6	61 (46-74)	60 (46-72)
IL-5 (> 2.0 pg/mL)	0.6	68 (52-80)	53 (39-67)
PDGF (> 347 pg/mL)	0.6	33 (20-47)	88 (76-94)
FGF-basic (> 2.0 pg/mL)	0.6	78 (63-88)	42 (29-56)
IL-2 (> 3.0 pg/mL)	0.6	32 (20-47)	84 (71-92)
IL-1β (> 0.3 pg/mL)	0.6	63 (47-76)	53 (40-66)
CCL5 (> 1,352 pg/mL)	0.6	35 (22-50)	78 (65-87)
IL-12 (> 0.5 pg/mL)	0.6	95 (83-99)	18 (10-31)
CCL11 (> 24 pg/mL)	0.5	58 (42-71)	59 (45-72)
IL-9 (> 8.0 pg/mL)	0.5	74 (59-85)	40 (28-54)
GM-CSF (< 0.3 pg/mL)	0.5	50 (34-66)	61 (44-76)
IL-13 (> 0.5 pg/mL)	0.5	73 (57-84)	37 (25-51)

AUC, Area Under the Receiver Operating Characteristic (ROC) curve; Se, Sensitivity; Sp, Specificity; IQR, Interquartile range. Immune mediators with AUC>0.8 are underscored by bold format.

## Discussion

This study was designed as an exploratory follow-up investigation to characterize the timeline kinetics and network interplay of serum immune mediators in COVID-19 patients, further categorized according to disease outcome.

At hospital admission, COVID-19 patients exhibited a broad inflammatory response characterized by a classical immune mediator storm, with prominent increases of chemokines and pro-inflammatory cytokines. In this study, we identified a set of eight mediators (CXCL8, CCL3, CCL5, CXCL10, IL-1β, IL-6, IFN-γ, and IL-1Ra) showing the most pronounced differences in COVID-19 patients as compared to healthy controls. These findings align with previous studies demonstrating that COVID-19 induces an exacerbated production of soluble immune mediators, recognized as central drivers of immunopathogenesis and adverse clinical outcomes (9–11). It is well established that the hyperactivation of pro-inflammatory pathways is a hallmark of COVID-19 and contributes to multiorgan dysfunction and increased mortality in a subset of patients (12, 13).

Extending these observations over time, our findings revealed distinct patterns throughout the timeline kinetics. While elevated levels of serum mediators were observed in COVID-19 patients, those who progressed to death showed higher concentrations at hospital admission and during follow-up. Longitudinal follow-up analysis further revealed that the “death” subgroup exhibited a persistent elevation of immune mediators across all timepoints, with higher imbalance at D8-14. This persistent inflammatory state reinforces the concept that sustained immune activation underlies unfavorable clinical evolution in COVID-19 (8, 14).

Overall, our data analysis demonstrated that a set of five immune mediators (CXCL8, CCL2, CXCL10, IL-6, and IFN-γ) showed consistently higher levels in the “death” subgroup. This chemokine profile aligns with previous studies showing that single-cell RNA sequencing analyses of bronchoalveolar lavage fluid (BALF) from COVID-19 patients revealed expansion of inflammatory macrophage subsets expressing high levels of CCL2 and CXCL10, underscoring their pivotal role in sustaining systemic inflammation and contributing to disease severity (15, 16). Similarly, neutrophils have emerged as major effector cells in COVID-19 immunopathology, particularly through CXCL8-mediated recruitment and activation. Moreover, single-cell transcriptomic analyses of BALF revealed elevated CXCL8 expression in both epithelial and myeloid compartments of critically ill patients, a key chemoattractant driving massive neutrophil infiltration into inflamed pulmonary tissues (17, 18). Furthermore, activated neutrophils display impaired phagocytic function and a hyperactivated phenotype, releasing neutrophil extracellular traps, reactive oxygen species, and proteolytic enzymes that synergistically amplify tissue injury and endothelial dysfunction in COVID-19 (17–20). In this study, elevated serum CXCL8 levels observed in patients who progressed to death reinforce the concept that CXCL8-driven neutrophil hyperactivation represents a key mechanism contributing to the amplified storm of immune mediators associated with COVID-19 outcomes. Altogether, these findings highlight a chemokine axis linking CXCL8, CCL2, and CXCL10, sustaining a self-perpetuating inflammatory loop that promotes adverse clinical outcomes.

Beyond chemokine-driven pathways, cytokine-mediated mechanisms also play a pivotal role in shaping disease progression. Among these, IL-6 and IFN-γ emerged as key regulators orchestrating systemic inflammation in COVID-19. IL-6 functions as a central amplifier of the inflammatory cascade in COVID-19, triggering acute-phase responses, promoting endothelial activation and increasing vascular permeability, which may lead to respiratory compromise and multiorgan injury (21, 22). Consistent with our findings, other studies have shown that higher IL-6 levels are strongly associated with mortality, underscoring its prognostic relevance and its role in linking early innate immune activation to the sustained inflammatory response that characterizes adverse outcomes in COVID-19 (23, 24). Similarly, IFN-γ acts as a critical mediator in COVID-19 immunopathogenesis, linking antiviral defense to inflammatory injury and amplifying immune activation. In addition, sustained IFN-γ signaling perturbs immune homeostasis by triggering the release of chemokines such as CXCL10 and CCL2 (25). CXCL10, also known as interferon-γ–inducible protein 10 (IP-10), is produced in response to interferon signaling, and persistent elevation of CXCL10 has been associated with sustained inflammatory responses and severe disease phenotypes in COVID-19 (8, 14). Besides, IFN-γ has also been identified as an independent biomarker of disease severity and mortality (26, 27). Therefore, IL-6 and IFN-γ establish a pro-inflammatory circuit that bridges innate and adaptive immune activation, thereby shaping the immunopathogenic landscape of severe COVID-19.

The integrative correlation network analysis revealed distinct immunological profiles associated with COVID-19 outcomes. The “death” subgroup exhibited a denser and more complex network, particularly with higher connectivity among chemokines, indicating a dysfunctional immune response, as previously described by Jardim-Santos et al. (2022). Conversely, the “discharge” subgroup presented a lower-density network, suggesting a controlled inflammatory response contributing to clinical recovery. In line with our findings, other studies reported a chemokine-enriched immune profile associated with death, highlighting the contribution of dysregulated inflammatory networks to disease severity (28). Similarly, previous studies have identified CXCL8 and IL-6 as central nodes within a highly interconnected immune network predictive of clinical progression (29). Taken together, these studies reinforce the utility of system-level network approaches to uncover immune signatures linked to COVID-19 prognosis. However, differences in network connectivity between COVID-19 patients according to disease outcome indicate that immune disorganization, rather than mediator abundance alone, is a key driver of poor outcomes. Altogether, our results support the notion that immunological network architecture, rather than isolated biomarker levels, provides valuable insights into disease trajectory, and may inform future strategies for early risk stratification in COVID-19.

To further investigate the discriminatory capacity of immune mediators to classify “death” from “discharge” outcome, we employed a decision-tree algorithm assembled from the five immune mediators that exhibited the highest overall accuracy in the single-parameter analysis. The decision-tree model identified IL-6 as the root attribute, followed by CCL2 and CXCL8 as branching nodes, accurately classifying patients according to disease outcome. These results demonstrate that integrating multiple soluble mediators within a hierarchical decision framework enhances the ability to discriminate subgroups, as compared with single-parameter analysis. This integrative systems biology approach reinforces the concept that immune mediator patterns, when analyzed collectively, can function as reliable classifiers of clinical trajectory, consistent with previous studies applying decision-tree and ROC-based strategies to predict COVID-19 severity.

It is well established that anti-inflammatory therapies administered to hospitalized COVID-19 patients may modulate circulating levels of chemokines, cytokines and growth factors. In the present study, all hospitalized COVID-19 patients were treated uniformly with a therapeutic regimen consisting of corticosteroids, antibiotics, and anticoagulants, regardless of their clinical progression to mechanical ventilation or death. This standardized therapeutic approach across the cohort minimizes the likelihood that the differences observed in serum levels of soluble immune mediators between the COVID subgroups are confounded by treatment variability. Thus, immune signatures identified in this study likely reflect intrinsic host response dynamics associated with disease severity, and outcome, rather than the differential effect of therapeutic interventions. Additional laboratory parameters including blood cell counts, acute phase proteins and SARS-CoV-2 viral load further substantiated the immunopathological profile observed in the “death” subgroup. In addition to soluble immune mediators, classical hematological alterations associated with COVID-19 severity were observed in patients who progressed to death, including lymphopenia, neutrophilia, an increased NLR, and CRP (1, 2, 5). Although the study was not designed to focus on hematological parameters, these findings support the presence of systemic immune dysregulation and are consistent with the severe clinical progression observed in this subgroup. Moreover, in the present study, AKI was more frequent in the “death” subgroup, while baseline CKD did not differ significantly between outcome subgroups. This finding suggests that AKI may reflect acute organ dysfunction driven by severe systemic inflammation and multi-organ failure, representing a marker of acute critical illness rather than baseline CKD in hospitalized COVID-19 patients.

This study presents limitations that should be acknowledged. First, the relatively small sample size, particularly the limited number of deceased patients compared to survivors, may reduce the statistical power and generalizability of findings. The observational design and the absence of adjustment for potential confounding factors, such as comorbidities, represent additional limitations. Moreover, the incomplete longitudinal follow-up of all participants hindered a more comprehensive temporal analysis of immune mediator dynamics. In addition, the use of variable temporal windows, inherent to a real-life hospital-based design during the early phase of the pandemic, may limit the temporal resolution of cytokine kinetics and should be considered when interpreting the dynamics of the immune mediator storm. Finally, further investigations are needed to elucidate the influence of sex, aging, and comorbidities on the immunological profiles associated with COVID-19 outcomes. It is also important to mention that our study was carried out during the circulation of the SARS-CoV-2 B.1.1.28 and B.1.1.33 strains and it is plausible that infections with other variants may elicit distinct immunological responses. According to the reports from the European Centre for Disease Prevention and Control (ECDC), the Alpha variant predominates in 2020, followed by VOI Delta, VOI Zeta (P.2), VOC Gamma (P.1), and later by VOI Omicron, which became dominant from January 2022 (30). These variants were characterized by specific mutations, particularly in the spike domain, associated with increased transmission patterns, distinct immune response profiles, and, in some cases, higher clinical severity. The predominant circulating variants represent a critical factor to be considered when investigating the immune response profile, as certain VOIs are linked to increased risk of reinfection and or more severe disease outcomes (31, 32). There is evidence that COVID-19 outcome was worse during the Delta and Omicron waves than in preceding periods (33). In this sense, the overall interpretation of the results obtained in this study should be performed cautiously in other epidemiological scenarios, since distinct circulating VOI would lead to distinct soluble immune mediator responses as previously reported.

Collectively, our findings highlight that COVID-19 outcomes are not only dictated by the magnitude of systemic inflammation, but rather by the persistence and hierarchical coordination of key immune mediators shaping disease trajectory. The decision-tree model identified IL-6, CCL2, and CXCL8 as pivotal biomarkers, delineating a predictive axis that integrates cytokine and chemokine responses underlying unfavorable clinical evolution. IL-6 emerged as the root attribute, reflecting its role as a central amplifier of inflammatory cascades, while CCL2 and CXCL8 acted as branching nodes associated with monocyte, and neutrophil recruitment, respectively. This triad reflects key mechanisms of COVID-19 immunopathogenesis, linking sustained cytokine amplification to chemokine and tissue injury. Therefore, the combined assessment of these soluble mediators provides a powerful framework for early risk stratification and precision prognostication, offering potential guidance for immune monitoring and targeted immunomodulatory interventions in severe COVID-19.

## Data Availability

The original contributions presented in the study are included in the article/**Supplementary Material**. Further inquiries can be directed to the corresponding authors.

## References

[1] Celik Tellioglu E Oncul A Diktas H Atasoy Tahtasakal C Aktas E Genc Yaman I . The role of dynamic changes in hematologic and biochemical parameters in predicting mortality in covid-19 patients. Sisli Etfal Hastan Tıp Bul. (2024) 58:371–80. doi: 10.14744/SEMB.2024.26096, PMID: 39411033 PMC11472194

[2] Setiati S Harimurti K Safitri ED Ranakusuma RW Saldi SRF Azwar MK . Risk factors and laboratory test results associated with severe illness and mortality in COVID-19 patients: A systematic review. Acta Med Indones. (2020) 52:227–45. 33020334

[3] Hiti L Markovič T Lainscak M Farkaš Lainščak J Pal E Mlinarič-Raščan I . The immunopathogenesis of a cytokine storm: The key mechanisms underlying severe COVID-19. Cytokine Growth Factor Rev. (2025) 82:1–17. doi: 10.1016/j.cytogfr.2024.12.003, PMID: 39884914

[4] Rosyid AN Puspitasari AD Sensusiati AD Nugraha J Amin M . Interleukin-6 and interleukin-10 as a predictor of mortality in elderly with COVID-19. Ann Afr Med. (2024) 23:575–9. doi: 10.4103/aam.aam_1_24, PMID: 39138918 PMC11556489

[5] Dos Santos Medeiros SMFR Sousa Lino BMN Perez VP Sousa ESS Campana EH Miyajima F . Predictive biomarkers of mortality in patients with severe COVID-19 hospitalized in intensive care unit. Front Immunol. (2024) 15:1416715. doi: 10.3389/fimmu.2024.1416715, PMID: 39281667 PMC11401048

[6] Zanza C Romenskaya T Manetti AC Franceschi F La Russa R Bertozzi G . Cytokine storm in COVID-19: immunopathogenesis and therapy. Med (Kaunas). (2022) 58:144. doi: 10.3390/medicina58020144, PMID: 35208467 PMC8876409

[7] Ghaffarpour S Ghazanfari T Ardestani SK Naghizadeh MM Vaez Mahdavi MR Salehi M . Cytokine profiles dynamics in COVID-19 patients: a longitudinal analysis of disease severity and outcomes. Sci Rep. (2025) 15:14209. doi: 10.1038/s41598-025-98505-y, PMID: 40269030 PMC12019550

[8] Cheng J Wang H Li C Yu J Zhu M . Characteristics of cytokines/chemokines associated with disease severity and adverse prognosis in COVID-19 patients. Front Immunol. (2024) 15:1464545. doi: 10.3389/fimmu.2024.1464545, PMID: 39654886 PMC11625740

[9] Almada L Angiolini SC Dho ND Dutto J Gazzoni Y Manzone-Rodríguez C . Different cytokine and chemokine profiles in hospitalized patients with COVID-19 during the first and second outbreaks from Argentina show no association with clinical comorbidities. Front Immunol. (2023) 14:1111797. doi: 10.3389/fimmu.2023.1111797, PMID: 36817433 PMC9929547

[10] Hu B Huang S Yin L . The cytokine storm and COVID-19. J Med Virol. (2021) 93:250–6. doi: 10.1002/jmv.26232, PMID: 32592501 PMC7361342

[11] Del Valle DM Kim-Schulze S Huang HH Beckmann ND Nirenberg S Wang B . An inflammatory cytokine signature predicts COVID-19 severity and survival. Nat Med. (2020) 26:1636–43. doi: 10.1038/s41591-020-1051-9, PMID: 32839624 PMC7869028

[12] Ghofrani Nezhad M Jami G Kooshkaki O Chamani S Naghizadeh A . The role of inflammatory cytokines (Interleukin-1 and interleukin-6) as a potential biomarker in the different stages of COVID-19 (Mild, severe, and critical). J Interferon Cytokine Res. (2023) 43:147–63. doi: 10.1089/jir.2022.0185, PMID: 37062817

[13] Hawerkamp HC Dyer AH Patil ND McElheron M O’Dowd N O’Doherty L . Characterisation of the pro-inflammatory cytokine signature in severe COVID-19. Front Immunol. (2023) 14:1170012. doi: 10.3389/fimmu.2023.1170012, PMID: 37063871 PMC10101230

[14] Schrijver B Assmann JLJC van Gammeren AJ Vermeulen RCH Portengen L Heukels P . Extensive longitudinal immune profiling reveals sustained innate immune activation in COVID-19 patients with unfavorable outcome. Eur Cytokine Netw. (2020) 31:154–67. doi: 10.1684/ecn.2020.0456, PMID: 33648924 PMC7937051

[15] Wei R Qin Z Huang Q Liu L Cheng F Meng S . A landscape study on COVID-19 immunity at the single-cell level. Front Immunol. (2022) 13:918383. doi: 10.3389/fimmu.2022.918383, PMID: 35911765 PMC9334848

[16] Liao M Liu Y Yuan J Wen Y Xu G Zhao J . Single-cell landscape of bronchoalveolar immune cells in patients with COVID-19. Nat Med. (2020) 26:842–4. doi: 10.1038/s41591-020-0901-9, PMID: 32398875

[17] Asaba CN Bitazar R Labonté P Bukong TN . Bronchoalveolar lavage single-cell transcriptomics reveals immune dysregulations driving COVID-19 severity. PloS One. (2025) 20:e0309880. doi: 10.1371/journal.pone.0309880, PMID: 39928675 PMC11809808

[18] Park JH Lee HK . Re-analysis of single cell transcriptome reveals that the NR3C1-CXCL8-neutrophil axis determines the severity of COVID-19. Front Immunol. (2020) 11:2145. doi: 10.3389/fimmu.2020.02145, PMID: 32983174 PMC7485000

[19] Li S Wang H Shao Q . The central role of neutrophil extracellular traps (NETs) and by-products in COVID-19 related pulmonary thrombosis. Immun Inflammation Dis. (2023) 11:e949. doi: 10.1002/iid3.949, PMID: 37647446 PMC10461423

[20] Veras FP Pontelli MC Silva CM Toller-Kawahisa JE de Lima M Nascimento DC . SARS-CoV-2-triggered neutrophil extracellular traps mediate COVID-19 pathology. J Exp Med. (2020) 217:e20201129. doi: 10.1084/jem.20201129, PMID: 32926098 PMC7488868

[21] Shekhawat J Gauba K Gupta S Purohit P Mitra P Garg M . Interleukin-6 perpetrator of the COVID-19 cytokine storm. Indian J Clin Biochem. (2021) 36:440–50. doi: 10.1007/s12291-021-00989-8, PMID: 34177139 PMC8216093

[22] Gubernatorova EO Gorshkova EA Polinova AI Drutskaya MS . IL-6: relevance for immunopathology of SARS-cov-2. Cytokine Growth Factor Rev. (2020) 53:13–24. doi: 10.1016/j.cytogfr.2020.05.009, PMID: 32475759 PMC7237916

[23] Nguyen CV Luong CQ Dao CX Nguyen MH Pham DT Khuat NH . Predictive validity of interleukin 6 (IL-6) for the mortality in critically ill COVID-19 patients with the B.1.617.2 (Delta) variant in Vietnam: a single-centre, cross-sectional study. BMJ Open. (2024) 14:e085971. doi: 10.1136/bmjopen-2024-085971, PMID: 39653572 PMC11628983

[24] Avila-Nava A Cortes-Telles A Torres-Erazo D López-Romero S Chim Aké R Gutiérrez Solis AL . Serum IL-6: A potential biomarker of mortality among SARS-CoV-2 infected patients in Mexico. Cytokine. (2021) 143:155543. doi: 10.1016/j.cyto.2021.155543, PMID: 33896708 PMC8052471

[25] Zhang F Mears JR Shakib L Beynor JI Shanaj S Korsunsky I . IFN-γ and TNF-α drive a CXCL10+ CCL2+ macrophage phenotype expanded in severe COVID-19 lungs and inflammatory diseases with tissue inflammation. Genome Med. (2021) 13:64. doi: 10.1186/s13073-021-00881-3, PMID: 33879239 PMC8057009

[26] Mansoor S Butt AR Bibi A Mushtaq S Ullah I Alshahrani F . Expression of IFN-Gamma is significantly reduced during severity of covid-19 infection in hospitalized patients. PloS One. (2023) 18:e0291332. doi: 10.1371/journal.pone.0291332, PMID: 37756264 PMC10530045

[27] Gadotti AC de Castro Deus M Telles JP Wind R Goes M Garcia Charello Ossoski R . IFN-γ is an independent risk factor associated with mortality in patients with moderate and severe COVID-19 infection. Virus Res. (2020) 289:198171. doi: 10.1016/j.virusres.2020.198171, PMID: 32979474 PMC7510544

[28] Jardim-Santos GP Schulte HL Kurizky PS Gomes CM Nóbrega OT de Gois ET . Unbalanced networks and disturbed kinetics of serum soluble mediators associated with distinct disease outcomes in severe COVID-19 patients. Front Immunol. (2022) 13:1004023. doi: 10.3389/fimmu.2022.1004023, PMID: 36451835 PMC9701840

[29] Liu Y Chen D Hou J Li H Cao D Guo M . An inter-correlated cytokine network identified at the center of cytokine storm predicted COVID-19 prognosis. Cytokine. (2021) 138:155365. doi: 10.1016/j.cyto.2020.155365, PMID: 33246770 PMC7651249

[30] European Centre for Disease Prevention and Control (ECDC) . SARS-CoV-2 variants of concern as of [data]. Available online at: https://www.ecdc.europa.eu/en/covid-19/variants-concern (Accessed September 18, 2025).

[31] Vieira DFB Bandeira DM da Silva MAN de Almeida ALT Araújo M MaChado AB . Comparative analysis of SARS-CoV-2 variants Alpha (B.1.1.7), Gamma (P.1), Zeta (P.2) and Delta (B.1.617.2) in Vero-E6 cells: ultrastructural characterization of cytopathology and replication kinetics. Braz J Infect Dis. (2024) 28:103706. doi: 10.1016/j.bjid.2023.103706, PMID: 38081327 PMC10776915

[32] Giovanetti M Slavov SN Fonseca V Wilkinson E Tegally H Patané JSL . Genomic epidemiology of the SARS-CoV-2 epidemic in Brazil. Nat Microbiol. (2022) 7:1490–500. doi: 10.1038/s41564-022-01191-z, PMID: 35982313 PMC9417986

[33] Male V . SARS-CoV-2 infection and COVID-19 vaccination in pregnancy. Nat Rev Immunol. (2022) 22:277–82. doi: 10.1038/s41577-022-00703-6, PMID: 35304596 PMC8931577

