# Supplementary material for: Exploring the timeline and network interplay of immune mediators in COVID-19 patients according to disease outcome
Source: Front Immunol. 2026 Mar 16;17:1765997. doi: 10.3389/fimmu.2026.1765997 (PMC13033563; doi:10.3389/fimmu.2026.1765997)
Supplement: Supplementary file 1 [file DataSheet1.pdf]

Supplementary Table 1. Laboratory records of COVID-19 patients at hospital admission classified according to disease outcome

Parameters	COVID (n=92)	COVID subgroups		
		Discharge (n=51)	Death (n=41)	p-Value
Hemoglobin (g/dL)	10.5 ± 2.6	10.3 ± 2.5	10.7 ± 2.8	0.5
Hematocrit (%)	32.1 ± 7.8	31.6 ± 7.1	32.5 ± 8.5	0.6
RDW (%)	16.0 ± 3.4	16.0 ± 3.2	16.0 ± 3.5	0.8
WBC ($x10^3/mm^3$)	8.7 ± 5.6	**7.2 ± 4.4**	**10.5 ± 6.3**	**0.002**
RBC ($x10^3/mm^3$)	3.7 ± 0.9	3.7 ± 0.8	3.7 ± 0.9	0.7
Platelets ($x10^3/mm^3$)	239.8 ± 115.1	242.6 ± 106.2	236.4 ± 126.2	0.5
Lymphocytes ($x10^3/mm^3$)	1.1 ± 0.7	1.1 ± 0.7	1.1 ± 0.7	0.9
Neutrophils ($x10^3/mm^3$)	7.0 ± 5.5	**5.8 ± 5.0**	**8.8 ± 5.6**	**0.001**
Band Cells ($x10^3/mm^3$)	0.7 ± 0.6	**0.5 ± 0.5**	**1.0 ± 0.6**	**0.0001**
Monocytes ($x10^3/mm^3$)	0.5 ± 0.4	0.5 ± 0.3	0.6 ± 0.4	0.5
Neu/Lym Ratio (NLR)	7.7 ± 5.9	**6.2 ± 4.5**	**9.4 ± 7.0**	**0.007**
Mon/Lym Ratio (MLR)	0.5 ± 0.3	0.5 ± 0.2	0.5 ± 0.3	0.8
D-dimer (ng/mL)	2,660 ± 2,505	2,409 ± 2,471	2,981 ± 2,550	0.1
C-reactive protein (mg/dL)	15.3 ± 13.1	**11.6 ± 10.7**	**19.6 ± 14.4**	**0.004**
Ferritin (ng/mL)	1,866 ± 2,054	1,725 ± 1,828	2,060 ± 2,354	0.5
Viral load (log_{10}copies/mL; mean ± SD)	3.4 ± 1.7	**2.9 ± 1.7**	**4.0 ± 1.5**	**0.002**

Data is presented as mean ± SD. Comparative analysis between Discharge vs Death was performed by Mann-Whitney test. WBC= White blood cells; RBC= red blood cells; RDW= red cell distribution width; NLR= neutrophil-lymphocyte ratio; MLR= monocyte-lymphocyte ratio. Reference ranges for Healthy Controls: Hemoglobin: 12-16 g/dL; Hematocrit: 36-46%; RDW: 11-16%; WBC: 4.5-10.5 $x10^3/mm^3$; RBC: 4.6-6.2 $x10^3/mm^3$; Platelets: 150-400 $x10^3/mm^3$; Lymphocytes: 0.8-4.0 $x10^3/mm^3$; Neutrophils: 1.8-7.0 $x10^3/mm^3$; Band cells: 0.0-0.2 $x10^3/mm^3$; Monocytes: 0.1-1.0 $x10^3/mm^3$; NLR: 1.0-3.0; MLR: 0.1-0.6; D-dimer: <500 ng/mL; C-reactive protein: 0.3-1.0 mg/dL; Ferritin: <350 ng/mL. Laboratory records of COVID group outside the reference ranges for healthy subjects are highlighted by underline format. Comparative analysis between Death vs Discharge was performed by t-student or Mann-Whitney test. Significant differences at $p<0.05$ are underscored by bold format.
