# Supplementary material for: Exploring the timeline and network interplay of immune mediators in COVID-19 patients according to disease outcome
Source: Front Immunol. 2026 Mar 16;17:1765997. doi: 10.3389/fimmu.2026.1765997 (PMC13033563; doi:10.3389/fimmu.2026.1765997)
Supplement: Supplementary file 2 [file DataSheet2.pdf]

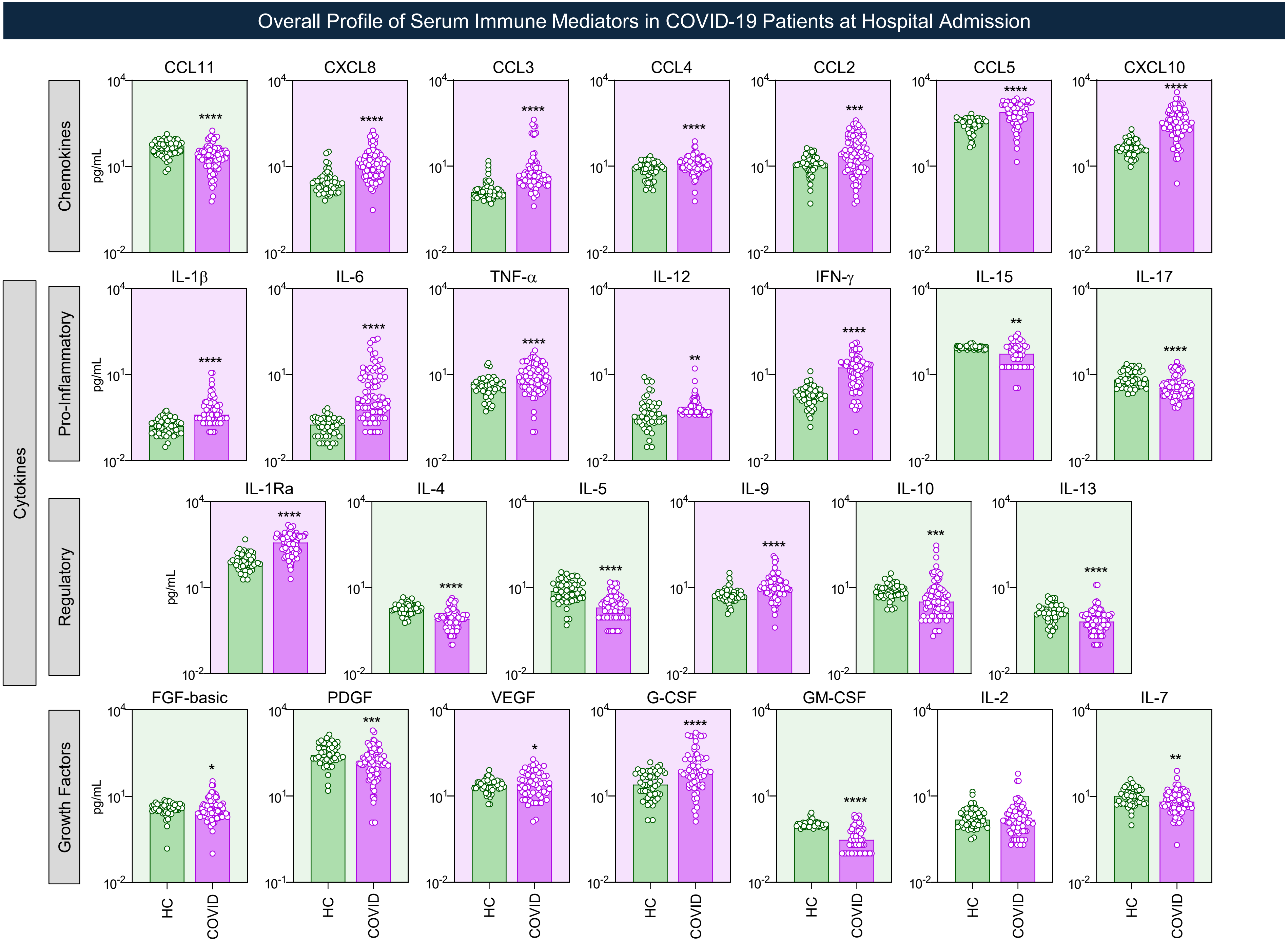

Supplementary Figure 1

Supplementary Figure 1. Overall profile of serum immune mediators in COVID-19 patients at hospital admission. The overall profile of chemokines, pro-inflammatory cytokines, regulatory cytokines and growth factors was evaluated in serum samples from COVID-19 patients at hospital admission (, COVID, n = 92) and compared to Healthy Controls (, HC, n = 50). Measurements of immune mediators were carried out by Luminex Bio-plex platform as described in Material and methods section. The results are presented as the scattering distribution of individual values over bars underscoring the median values of serum immune mediators. Comparative analysis between HC vs COVID was performed by the Mann-Whitney test. Significant differences are represented by *, **, *** or **** to denote p values <0.05, <0.01, <0.001 or <0.0001, respectively. Background colors were used to underscore the serum immune mediators with increased (purple) or decreased (green) levels in COVID group as compared to HC.
