# Supplementary material for: Exploring the timeline and network interplay of immune mediators in COVID-19 patients according to disease outcome
Source: Front Immunol. 2026 Mar 16;17:1765997. doi: 10.3389/fimmu.2026.1765997 (PMC13033563; doi:10.3389/fimmu.2026.1765997)
Supplement: Supplementary file 3 [file DataSheet3.pdf]

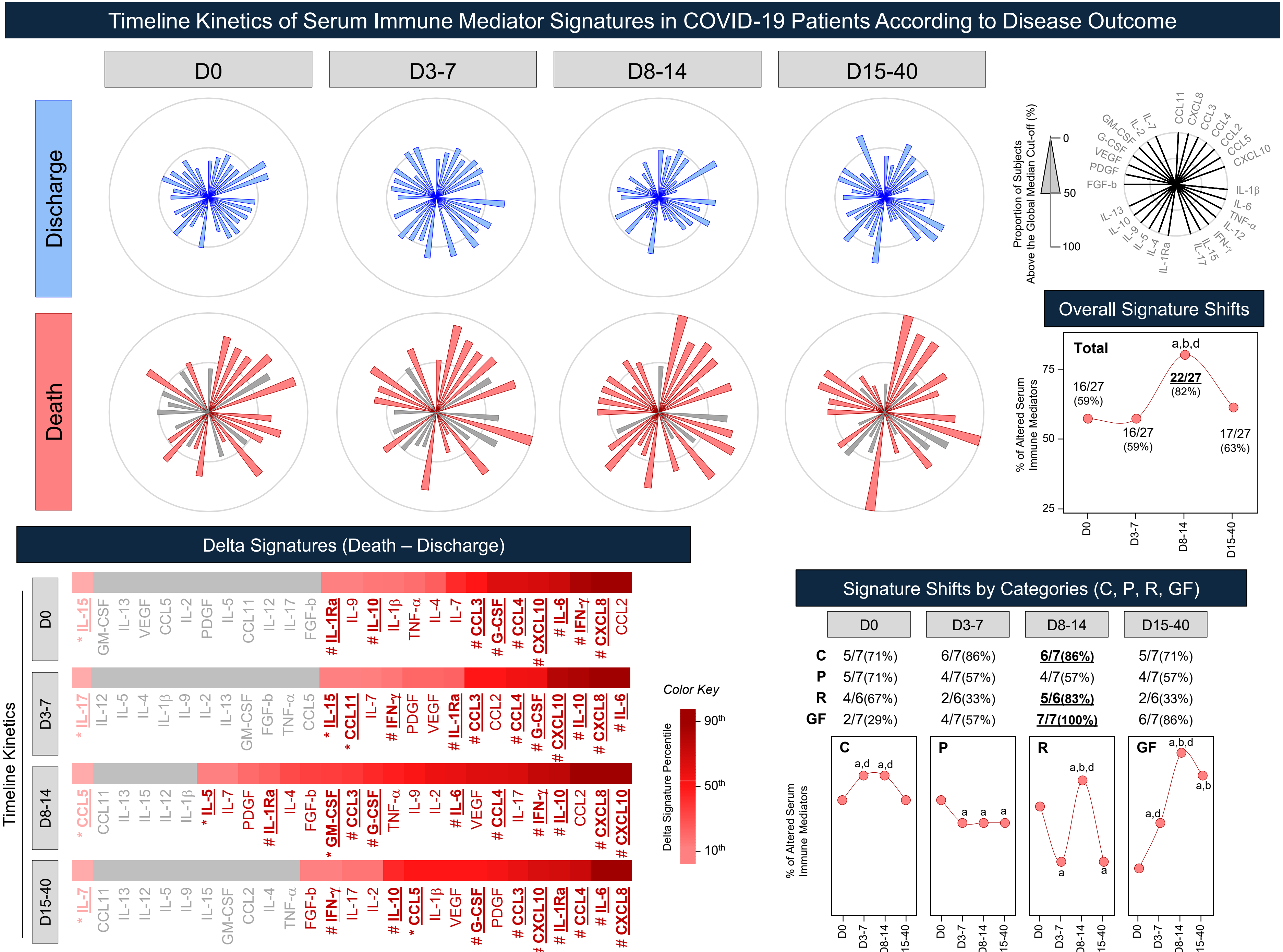

Supplementary Figure 2

Supplementary Figure 2. Timeline kinetics of serum immune mediator signatures in COVID-19 patients according to disease outcome. The overall signature of chemokines, pro-inflammatory cytokines, regulatory cytokines and growth factors were assembled for serum samples from COVID-19 patients at consecutive timepoints (D0, D3-7, D8-14 and D15-40). COVID-19 patients were further categorized according to disease outcome [Discharge (, Discharge, n = 51) and Death (, Death, n = 41)]. Measurements of serum mediators were carried out by Luminex Bio-plex platform as described in Material and methods section. Data are shown in radar charts representing the proportion of subjects with serum levels above the global median cut-off (%), calculated for each immune mediator. Comparative analysis between Discharge and Death subgroups was carried out by Fisher test. Significant differences ($p < 0.05$) in Death subgroup in comparison to Discharge subgroup are underscored by red radar bars. Non-significant differences are represented by gray radar bars. Changes in immune mediator signatures were calculated as “Delta Signature = % in Death – % in Discharge” along the timeline kinetics and represented by colormaps. Common and selective serum immune mediators are identified by # and * symbols, respectively. A color key was applied to grade the delta signature values. Line charts represent the shift in delta signature and reported as % of altered immune mediators (overall and by categories). Significant differences ($p < 0.05$) in delta signature identified by Fisher test were underscored with letters “a”, “b” and “d” for comparisons with D0, D3-7 and D15-40, respectively.
